# Protein arginine methyltransferase 8 regulates ferroptosis and macrophage polarization in spinal cord injury via glial cell‐derived neurotrophic factor

**DOI:** 10.1111/cns.14162

**Published:** 2023-03-13

**Authors:** Zehua Zou, Ruixuan Liu, Yiwen Wang, Hongjian Tan, Gang An, Baifeng Zhang, Yongzhi Wang, Daming Dong

**Affiliations:** ^1^ Department of Orthopedics (Five) First Affiliated Hospital of Harbin Medical University Harbin P.R. China

**Keywords:** ferroptosis, GDNF, M1 macrophage polarization, PRMT8, spinal cord injury

## Abstract

**Objective:**

To explore the influence of protein arginine methyltransferase 8 (PRMT8) regulating glial cell‐derived neurotrophic factor (GDNF) on neuron ferroptosis and macrophage polarization in spinal cord injury (SCI).

**Methods:**

A rat model of SCI was established through an injury induced by an external force. Basso, Beattie, and Bresnahan score, hematoxylin and eosin staining, and immunofluorescence were used, respectively, to detect changes in rat locomotion, spinal cord histopathology, and NeuN expression in the spinal cord. Iron content in the spinal cord and levels of malondialdehyde and glutathione were measured using detection kits. Transmission electron microscopy was used to reveal the morphological characteristics of mitochondria. Western blotting was performed to detect PRMT8, GDNF, cystine/glutamate transporter XCT, glutathione peroxidase 4, 4‐hydroxynonenal, heme oxygenase‐1, inducible nitric oxide synthase (iNOS), CD16, and arginase 1 (Arg1). The expression levels of iNOS and Arg1 in the spinal cord were visualized by immunofluorescence. ELISA was performed to measure the expression levels of IL‐6, IL‐1β, and TNF‐α. Rat dorsal root ganglion (DRG) neurons and RMa‐bm rat macrophages were treated with lipopolysaccharide under hypoxic conditions. The viability and iron content of the neurons were detected using Cell Counting Kit‐8 and a specific probe, respectively. Flow cytometry and immunofluorescence were used to assess macrophage polarization. Chromatin immunoprecipitation was used to identify the binding of PRMT8 to the *GDFN* promoter.

**Results:**

Neuronal ferroptosis and M1 macrophage polarization were promoted, and PRMT8 expression was downregulated in SCI. PRMT8 overexpression exerted therapeutic effects on injured DRG neurons and RMa‐bm cells. Moreover, PRMT8 overexpression inhibited ferroptosis and M1 macrophage polarization in rats with SCI. PRMT8 promoted GDNF expression by catalyzing H3K4 methylation. Knockdown of *GDNF* counteracted the therapeutic effects of PRMT8 overexpression.

**Conclusion:**

Overexpression of PRMT8 may inhibit ferroptosis and M1 macrophage polarization by increasing GDNF expression, thereby alleviating SCI.

## Graphical abstract

Low PRMT8 expression inhibits GDNF transcriptional expression by reducing H3K4 methylation, thereby promoting ferroptosis and M2 macrophage polarization in SCI rats.

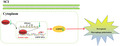

## 1 INTRODUCTION

Spinal cord injuries (SCIs) are incidents of devastating damage to the spinal cord resulting from either physical trauma or non‐traumatic causes, which impose serious health and financial burdens on each patient.^1^ SCI occurs in two phases: primary and secondary injuries. The primary injury includes hemorrhage and the destruction of the neural parenchyma, axonal network, and glial membranes. These events trigger secondary injury, which causes further chemical and mechanical damage to spinal tissues.^2^ Recent research on the treatment of SCI has focused on reducing secondary injury and enhancing neural regeneration.^3^

Ferroptosis is an iron‐dependent form of regulated cell death caused by the accumulation of lipid‐based reactive oxygen species (ROS), which is distinct from other forms of regulated cell death.^4^ Cell death is responsible for functional deterioration after SCI, and many forms of cell death, such as apoptosis, necroptosis, autophagy, and ferroptosis, are involved.^5^ Inflammation also contributes to the disruption of neuronal homeostasis and neurodegeneration in SCI. The cores of SCI lesions are infiltrated by blood‐derived monocytes/macrophages, which show activated phenotypes.^6^ Macrophages undergo phenotypic polarization to obtain the required functional phenotype (inflammatory M1 or anti‐inflammatory M2) in response to various environmental stimuli.^7^ Developing therapies to target molecules involved in ferroptosis and macrophage polarization could improve the outcomes of SCI.

Protein arginine methyltransferase 8 (PRMT8) is an enzyme that induces asymmetrical dimethylation of its substrates (to add two methyl groups on one of the guanidino nitrogen atoms), thereby regulating fundamental processes, such as RNA processing, chromatin organization, gene expression, and metabolism.^8^ PRMT8 is localized to the plasma membrane via N‐terminal myristoylation and is specifically expressed in the central nervous system.^9^ A recent study showed that knockout of *PRMT8* affected membrane phospholipid composition, reduced mitochondrial stress capacity, and increased neuroinflammatory markers in the brains of mice exposed to hypoxic stress.^10^ Therefore, we speculated that PRMT8, a neuroprotective molecule, might be downregulated in SCI.

Glial cell‐derived neurotrophic factor (GDNF), a member of the transforming growth factor‐β (TGF‐β) superfamily, has shown protective effects on specific neuronal populations.^11^ The binding of GDNF to its receptors activates several intracellular signaling pathways that promote the development and survival of neurons and maintain neuronal interactions with other neurons or target tissues.^12^ Owing to its neuroprotective ability, GDNF has been employed alone or in combination therapies to spare motor neurons from experimentally induced SCI.^13^ However, it remains unknown whether PRMT8 and GDNF interact to regulate neuronal function.

This study investigates the function of PRMT8 in ferroptosis and macrophage polarization in SCI and explores the interaction between PRMT8 and GDNF to identify new targets for SCI therapies.

## 2 MATERIALS AND METHODS

### 2.1 SCI model

Forty‐eight 8‐week‐old Sprague–Dawley rats were purchased from Hunan SJA Laboratory Animal Co., Ltd. (Hunan, China). All animal experiments followed the rules set by the Animal Experimentation Ethics Committee of the First Affiliated Hospital of Harbin Medical University and were approved by the committee. The experimental protocols were designed in accordance with *the Guidelines for the Care and Use of Laboratory Animals* issued by the National Institutes of Health (NIH).^14^

Dorsal laminectomy at the tenth thoracic vertebra (T10) was performed on anesthetized rats to expose the spinal dural sac.^15^ An infinite horizontal impactor (Precision Systems and Instrumentation) was used to generate a free fall from a height of 3 cm to cause a moderate contusion (with a force of 50 kdyn) to part of the spinal cord corresponding to the T10 bone window.^16^ The SCI model was considered successful when the following phenomena appeared: convulsive sways of the tail, quick retraction and sways of the lower limbs, edema and hemorrhage of the impacted spinal cord tissue, and intact and purple‐red spinal dura mater. For the sham operation, the region of the spinal cord corresponding to the T10 vertebra was exposed, but not hit. PRMT8 expression was increased by *PRMT8* overexpression lentiviruses (oe‐*PRMT8*), while GDNF expression was modified by *GDNF* overexpression (oe‐*GDNF*) or silencing (sh‐*GDNF*) lentiviruses. Empty lentiviral vectors were used as the negative controls (oe‐NC and sh‐NC). The 48 rats were divided into sham, SCI, SCI + oe‐NC, SCI + oe‐*PRMT8*, SCI + oe‐NC + sh‐NC, SCI + oe‐*PRMT8* + sh‐NC, SCI + oe‐*GDNF* + sh‐NC, and SCI + oe‐*PRMT8* + sh‐*GDNF* groups (six rats in each group). Lentiviruses (3 μL, 0.4 μL/min) were directly injected into the cavity of injured spinal cord using a Hamilton syringe, which was positioned using a stereotaxic apparatus (Kopf Instruments, Tujunga, CA, USA). After spinal cord contusion and lentiviral injection, the wound was washed with 0.9% sodium chloride solution and sutured layer‐by‐layer. After the operation, the rats were housed separately in cages and kept warm, and their bladders were manually massaged twice daily to help them defecate until spontaneous bowel movements were restored.

### 2.2 Basso, Beattie, and Bresnahan score

Open‐field locomotor testing was performed to assess the functional recovery of the rats on days 0, 7, 14, 21, and 28 after SCI modeling. The Basso, Beattie, and Bresnahan (BBB) score,^17^ ranging from 0 to 21, was used to quantify motor abilities, including angular movement range of three joints, weighted stepping, gait coordination, and tail movement. A score of 0 indicated complete paralysis and 21 indicated normal locomotion. BBB scores were given by two investigators blinded to the animal groupings.

### 2.3 Hematoxylin–eosin staining

Injured spinal cord segments were collected on the 28th day after SCI modeling, embedded in paraffin, and sliced continuously. Tissue sections (4 μm) were attached to slides in 46°C water and baked for 2 h in a 72°C oven. The sections were cooled for 10 min and sequentially soaked in the following solutions: xylene I (10 min), xylene II (10 min), absolute ethanol I (5 min), absolute ethanol II (5 min), 90% ethanol (2 min), 80% ethanol (2 min), 70% ethanol (2 min), running water (5 min), hematoxylin (5–10 min), running water (5 min), hydrochloric acid‐alcohol (2–3 s), running water (5 min), lithium carbonate (10 min), running water (10 min), eosin (2 min), running water (5 min), 80% ethanol (2 min), 90% ethanol (2 min), absolute ethanol (2 min), and xylene (2 min). Sections were mounted with neutral balsam and observed under a light microscope.

### 2.4 Immunofluorescence

Injured spinal cord segments were collected on the 28th day after SCI modeling, embedded in paraffin, and sliced continuously. Deparaffinized tissue sections were placed in citrate buffer and boiled for antigen retrieval (3 × 5 min) in a microwave oven. The sections were then washed three times with phosphate buffered saline (PBS) at room temperature for 3 min each. Cells collected at 24 h post‐lipopolysaccharide (LPS) stimulation or 72 h post‐transfection were fixed with 4% paraformaldehyde for 30 min, treated with 0.3% Triton X‐100 for 1 h, and soaked in 5% bovine albumin for 30 min. Tissue sections or cell slides were incubated with antibodies against F4/80 (1:200, ab300421, Abcam, Cambridge, UK), inducible nitric oxide synthase (iNOS; 1:200, ab15323, Abcam), arginase 1 (Arg1; 1:50, #93668, Cell Signaling Technology, Danvers, MA, USA), or NeuN (1:200, ab177487, Abcam). After washing with PBS three times, the samples were incubated with Cy3‐labeled goat anti‐rat IgG (H&L) (1:100, ab6953, Abcam) or FITC‐labeled goat anti‐rabbit IgG (H&L) (1:100, ab6717, Abcam) for 1 h at room temperature. The samples were counterstained with 4′,6‐diamidino‐2‐phenylindole for 20 min at room temperature away from light and mounted with an anti‐fluorescence quenching reagent. Protein expression was visualized under a fluorescence microscope (Olympus, Tokyo, Japan). For each slide, positive cells in five random fields of view (×200) were counted using ImageJ software (NIH).

### 2.5 Cell culture and grouping

Rat dorsal root ganglion (DRG) neurons (YS1005C) and RMa‐bm rat macrophages (YS1446C) (Shanghai Yaji Biotechnology Co., Ltd., Shanghai, China) were cultured in high‐glucose DMEM containing 10% fetal bovine serum (Thermo Fisher, Shanghai, China) at 37°C with 5% CO_2_. The adherent cells were passaged and digested with 0.25% trypsin (HyClone). Experiments were performed on cells in the logarithmic growth phase.

Neurons and macrophages were treated with 10 mg LPS under hypoxic conditions (2% O_2_) for 24 h to simulate acute SCI in vitro.^18^ Untreated cells were used as the controls. The treated cells were grouped as follows: treatment (LPS + hypoxia), treatment + oe‐NC, treatment + oe‐*PRMT8*, treatment + oe‐NC + sh‐NC, treatment + oe‐*PRMT8* + sh‐NC, treatment + oe‐*GDNF* + sh‐NC, and treatment + oe‐*PRMT8* + sh‐*GDNF*. Virus titers were determined using a p24 ELISA kit (Cell Biolabs, San Diego, CA, USA) 48 h after lentiviral transfection reagents were delivered into HEK293T cells. The virus titer was 4–5 × 10^8^ TU/mL in the experimental groups and 8 × 10^8^ TU/mL in the control group. Neurons and macrophages were infected with lentiviral particles and stimulated with LPS for 24 h and further cultured for 48 h before the selection of stable cell lines using puromycin (P8230, Solarbio, Beijing, China). Therefore, these cells were collected at 72 h post‐transfection for subsequent experiments. Nontransfected cells were collected for subsequent experiments after 24 h of LPS stimulation.

### 2.6 Quantitative reverse transcription polymerase chain reaction

Total RNA was extracted with an RNeasy Mini Kit (Qiagen, Valencia, CA, USA) from spinal cord tissues collected on the 28th day after SCI modeling and from cells harvested at 24 h post‐LPS stimulation or 72 h post‐transfection. A NanoDrop microvolume spectrophotometer was used to measure the purity and concentration of RNA. A reverse transcription kit was used to obtain cDNA. Quantitative polymerase chain reaction (qPCR) was performed using a SYBR® Premix Ex Taq™ II (Perfect Real Time) kit (DRR081, Takara, Toyko, Japan) and a real‐time fluorescence qPCR machine (ABI 7500, ABI, Foster City, CA, USA), with 3 replicates for each sample. The 2^−ΔΔCt^ method was adopted for data analysis. ΔΔCt = (Ct_target gene_ − Ct _reference GAPDH gene_)_experimental group_ − (Ct_target gene_ − Ct _reference GAPDH gene_)_control group_. PCR primers are as follows: PRMT8: forward primer, 5′‐CAGCGCAACGACTATGTCCA‐3′; reverse primer, 5′‐AGTGAGTGTAGGGGGCATCA‐3′. GDNF: forward primer, 5′‐ACACTCGAGGAGGAAGGACA‐3′; reverse primer, 5′‐CAATCGCAACTTGGTGACGG‐3′. GAPDH: forward primer, 5′‐GCATCTTCTTGTGCAGTGCC‐3′; reverse primer, 5′‐GATGGTGATGGGTTTCCCGT‐3′.

### 2.7 Western blot

Spinal cord tissues collected on the 28th day after SCI modeling or cells harvested at 24 h post‐LPS stimulation or 72 h post‐transfection were lysed with enhanced radioimmunoprecipitation assay (RIPA) buffer (Boster, Wuhan, China) containing protease inhibitors. Protein concentration was determined using a bicinchoninic acid (BCA) kit (Boster). Proteins after separation by 10% SDS‐PAGE were electroblotted onto a PVDF membrane, which was then placed in 5% BSA at room temperature for 2 h before incubation at 4°C overnight with diluted antibodies against PRMT8 (PA5‐120639, 1:1000; Thermo Fisher, Waltham, MA, USA), GDNF (ab119473, 1:2000), cystine/glutamate transporter (XCT; ab175186, 1:1000), glutathione peroxidase 4 (GPX4; ab125066, 1:1000), 4‐hydroxynonenal (4‐HNE; ab243070, 1:1000), iNOS (ab178945, 1:1000), CD16 (ab198507, 1:500), Arg1 (ab233548, 1:2000), heme oxygenase‐1 (HO‐1; ab68477, 1:500), H3K4me3 (ab8580, 1:500), lamin A (ab8980, 1:500), growth associated protein 43 (GAP43; ab8580, 1:100), neurofilament 200 (NF200; ab134306, 1:500), or GAPDH (ab8245, 1:5000) (All from Abcam). After washing, the membrane was incubated with HRP‐labeled rabbit anti‐mouse or goat anti‐rabbit secondary antibodies (ab6728, ab6721, respectively; 1:2000; Abcam) at room temperature for 1 h. The membrane was treated with ECL working reagent (EMD Millipore, Burlington, MA, USA) at room temperature for 1 min, after which excess ECL solution was removed. The membrane was sealed with a plastic wrap and exposed to an X‐ray film for 5–10 min. Protein expression was quantified using ImageJ software, with lamin A or GAPDH as the reference. The experiment was repeated three times.

### 2.8 Cell counting kit‐8 assay

Cells in the logarithmic growth phase were precultured in a 96‐well plate (1 × 10^4^ cells/well) for 24 h. After transfection, the cells were incubated with the cell counting kit‐8 (CCK‐8) reagent (10 μL per well; CK04, Dojindo, Kumamoto, Japan) at 37°C for 3 h. Absorbance was measured using a microplate at 450 nm to determine cell viability.

### 2.9 Flow cytometry

RMa‐bm cells collected 24 h after LPS stimulation or 72 h after transfection were trypsinized, washed, and resuspended in PBS (1 × 10^6^ cells). To detect cell polarization, 1 × 10^5^ RMa‐bm cells were resuspended in 100 μL PBS and incubated with 1 μL APC‐labeled anti‐iNOS (eBioscience, San Diego, CA, USA) and 1 μL PE‐labeled anti‐Arg1 (eBioscience) on ice for 30 min in the dark. The cells were then washed and resuspended in 300 μL PBS. The expression levels of iNOS and Arg1 were measured using flow cytometry.

### 2.10 Iron ion detection

On the 28th day after SCI modeling, the injured spinal cord tissue was isolated and weighed after cardiac perfusion with ice‐cold PBS. The tissue homogenate was added to 1 mL HCL (8.5 M) and hydrolyzed at 95°C for 1 h. After the sample was cooled to room temperature, 2 mL trichloroacetic acid (20%) was added to precipitate proteins. The sample was then centrifuged and the supernatant (220 μL) was mixed with 60 μL iron detection reagent (H_2_O solution containing 6.5 mM ferrozine, 6.5 mM neocupoline, 2.5 mM ammonium acetate, and 1 M ascorbic acid). After 30 min, the absorbance was measured at 562 nm and the iron concentration (μg/g spinal cord tissue) was calculated by comparison with the standard curve.

Neurons collected 24 h after LPS stimulation or 72 h after transfection were seeded in 24‐well plates. After reaching 60–70% confluence, the cells were incubated with 100 μL FeRhoNoxTM‐1 solution at 37°C for approximately 1 h. The cells were washed twice with Hanks' balanced salt solution and photographed under a fluorescence microscope in a randomized manner. The average intensity of red fluorescence was calculated using ImageJ software. The experiment was repeated three times.

### 2.11 Transmission electron microscopy

Spinal cord tissues collected on the 28th day after SCI modeling were cut into sections (70–90 nm) and fixed in 2% osmium tetroxide. The tissue sections were stained with 2% uranyl acetate, dehydrated in ethanol, and embedded in eponate. The sections were then placed on copper grids and stained with 2% uranyl acetate and lead citrate. Images were captured using transmission electron microscopy (TEM; 7600; Hitachi, Tokyo, Japan).

### 2.12 ELISA

The expression of TNF‐α, IL‐6, and IL‐1β in the rat spinal cord tissue collected on the 28th day after SCI modeling and macrophages after 24 h of LPS stimulation or at 72 h post‐transfection was detected using ELISA kits (96 T, Dakewe, Shenzhen, China). Malondialdehyde (MDA) and glutathione (GSH) levels in the rat spinal cord tissue collected on the 28th day after SCI modeling were measured using detection kits according to the manufacturer's instructions. Briefly, the sample (100 μL) was incubated with biotinylated antibody working solution (1:100, 100 μL/well) for 2 h. Optical density values were measured at 450 nm, and the results were calculated by comparison with standards and blanks. The experiments were repeated three times.

### 2.13 Chromatin immunoprecipitation

Cells were treated with 4% formaldehyde (final concentration: 1%) and sonicated. Anti‐H3K4me3 (ab8580, 1:50, Abcam, UK) was added to bind the *GDNF* promoter. Protein A Agarose/SaLmon Sperm DNA was used to precipitate the promoter complexes. The precipitated complex was washed to remove non‐specific binding. The *GDNF* promoter complex was obtained after elution and then decrosslinked. The *GDNF* promoter fragments were purified and detected by qPCR. The experiment was repeated three times.

### 2.14 Statistical analysis

SPSS21.0 software (SPSS Inc., Chicago, IL, USA) was used for the statistical analysis. Measurement data are expressed as the mean ± standard deviation. First, the skewness and kurtosis coefficients were used to test the normality of the data. Next, an independent samples *t*‐test or two‐way analysis of variance (ANOVA) was performed to compare the two groups of data. One‐way ANOVA was used for comparisons between multiple groups, followed by Tukey's multiple comparisons test. *p* < 0.05 indicated that the difference was significant.

## 3 RESULTS

### 3.1 Ferroptosis, M1 macrophage polarization, and low PRMT8 expression in SCI

**FIGURE 1:**
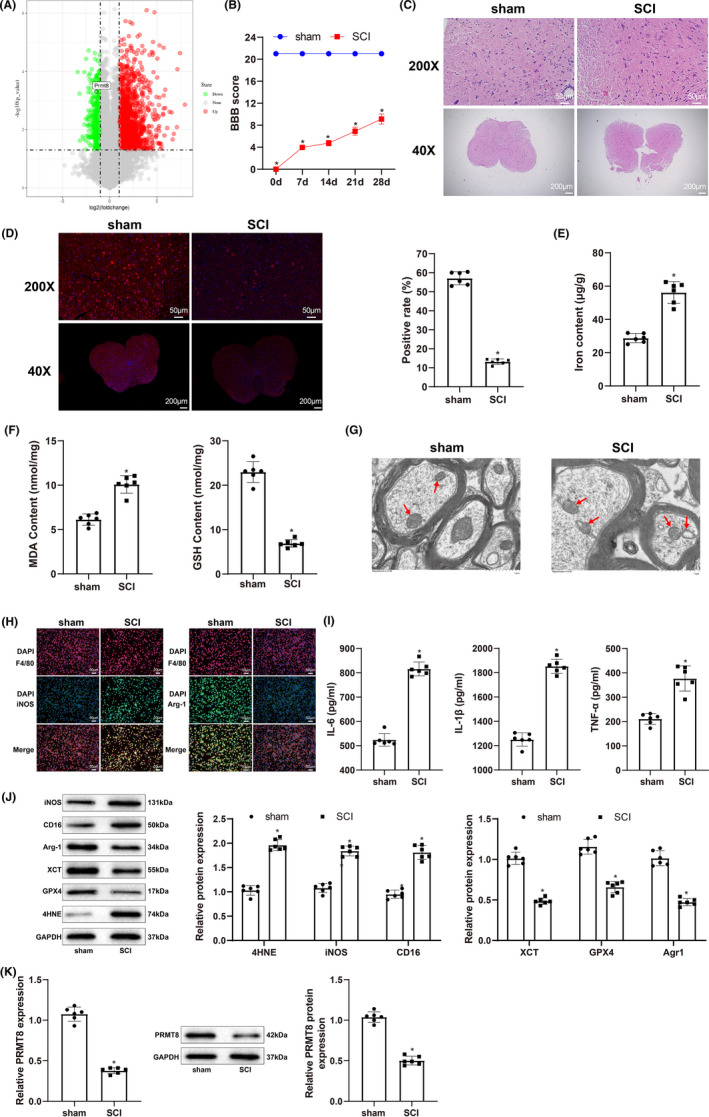
Ferroptosis, M1 macrophage polarization, and low PRMT8 expression in SCI. (A) Analysis of GSE166967 dataset in the GEO database for detecting PRMT8 expression. (B) BBB score for assessing locomotion. (C) H&E staining for histopathology examination of spinal cord. (D) Immunofluorescence for revealing NeuN expression in spinal cord. (E) Iron content in spinal cord. (F) Expression levels of MDA and GSH. (G) Transmission electron microscopy for examining the morphology of mitochondria in spinal cord (scale bar = 1.0 μm). (H) Immunofluorescence for revealing iNOS and Arg1 expression in spinal cord tissue. (I) ELISA for detecting the expression of IL‐6, IL‐1β and TNF‐α. (J) Western blot for detecting the expression of ferroptosis markers (XCT, GPX4, and 4‐HNE) and macrophage polarization markers (iNOS, CD16, and Agr1). (K) qRT‐PCR and western blot for detecting the expression of PRMT8. The data were expressed as mean ± standard deviation. Independent samples *t*‐test was used for comparisons between two groups. The BBB scores were compared by two‐way analysis of variance. *n* = 6. *p < 0.05 versus the sham group.

Through analysis of the GSE166967 dataset in the Gene Expression Omnibus database, we found that PRMT8 was expressed at low levels in the spinal cord of SCI mice (Figure 1A). To explore the role of PRMT8 in SCI, we established a rat SCI model and used the BBB score to assess locomotion. SCI rats had a lower BBB score than sham‐operated rats (Figure 1B). Histological examination of hematoxylin and eosin (H&E) staining showed that the spinal cord of SCI rats had damaged structures and unclear boundaries. Cells in the spinal cord were unevenly distributed and their nuclei were enlarged (Figure 1C). The expression of the neuronal marker NeuN, as revealed by immunofluorescence, was decreased in the spinal cord of SCI rats (Figure 1D). These results indicated that a rat model of SCI was successfully established. Next, we probed iron content and macrophage polarization in SCI. We found that, compared with sham‐operated rats, SCI rats had a higher iron content in the spinal cord (Figure 1E), a higher level of MDA, and a lower level of GSH (Figure 1F). The morphology of the mitochondria was examined by TEM. The images showed that the characteristics of ferroptosis, such as mitochondrial shrinkage, mitochondrial membrane thickening, and mitochondrial cristae loss, were present in SCI rats (Figure 1G). Immunofluorescence images of spinal cord tissue samples showed that the expression of the M1 macrophage marker iNOS increased, and that of the M2 macrophage marker Arg1 decreased in SCI rats (Figure 1H). The ELISA results showed that SCI rats had higher expression levels of IL‐6, IL‐1β, and TNF‐α than sham‐operated rats (Figure 1I). Western blotting was used to detect the expression of the ferroptosis markers XCT, GPX4, and 4‐HNE, as well as that of iNOS, CD16, and Arg1. Decreases in the expression of XCT, GPX4, and Arg1, and increases in the expression of 4‐HNE, iNOS, and CD16 were detected in SCI rats (Figure 1J). In addition, the expression of neurogenesis‐related proteins GAP43 and NF200 was also detected. The western blot results showed that the expression of GAP43 and NF200 in SCI rats was significantly lower than that in sham‐operated rats (Figure S1A). Consistent with public sequencing data, qRT‐PCR and western blotting analyses showed that the expression levels of PRMT8 mRNA and protein were reduced in SCI rats (Figure 1K). Taken together, these results indicate that ferroptosis and M1 macrophage polarization are promoted and PRMT8 is downregulated in SCI.

### 3.2 Overexpression of PRMT8 protects DRG neurons and regulates RMa‐bm polarization in vitro

**FIGURE 2:**
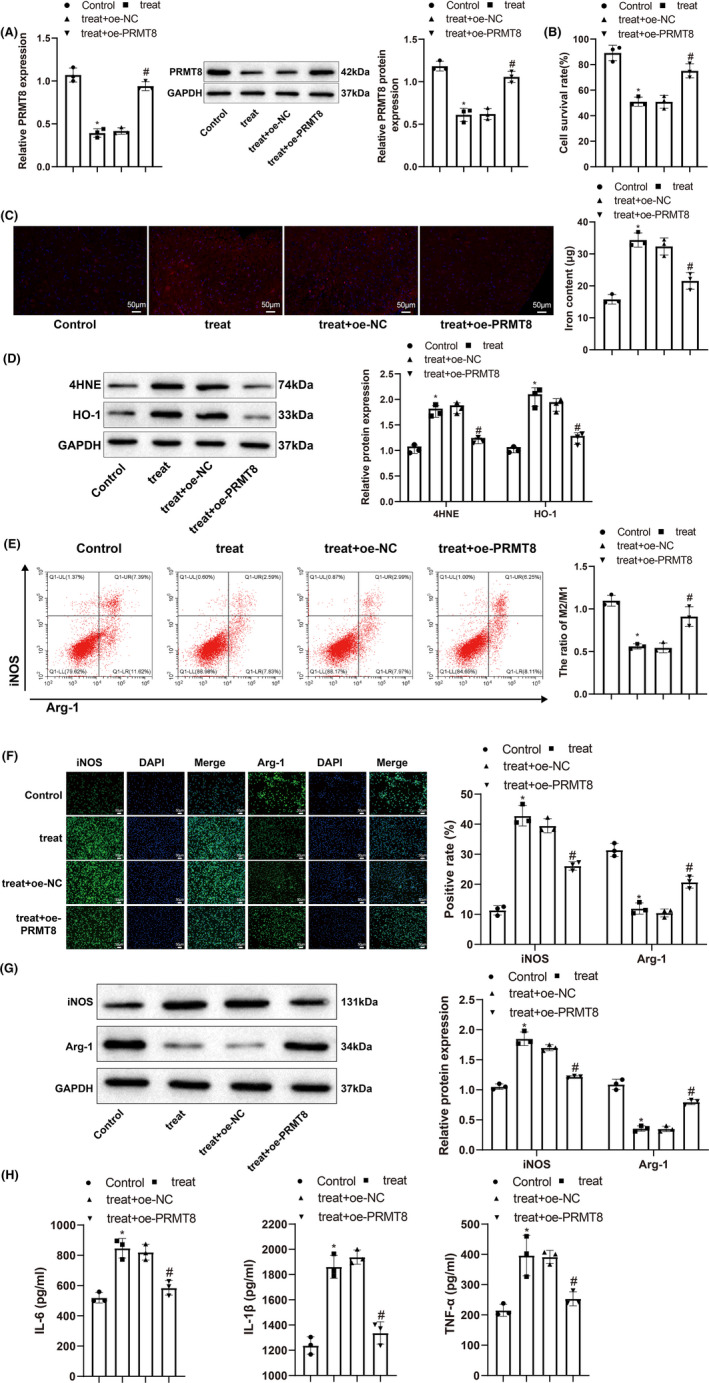
Overexpression of PRMT8 has therapeutic effects on DRG neurons and RMa‐bm in vitro. (A) qRT‐PCR and western blot for detecting the expression levels of PRMT8 mRNA and protein. (B) CCK‐8 for detecting the survival of DRG neurons. (C) Specific fluorescent probe FeRhoNoxTM‐1 for detecting the content of active iron in DRG neurons. (D) Western blot for detecting the expression levels of 4‐HNE and HO‐1 in DRG neurons. (E) Flow cytometry for detecting M1 and M2 macrophages. (F) Immunofluorescence for visualizing the expression of iNOS and Arg1 in macrophages. (G) Western blot for detecting the expression levels of iNOS and Arg1 in macrophages. (H) ELISA for measuring the expression levels of IL‐6, IL‐1β and TNF‐α in macrophages. The data were expressed as mean ± standard deviation. One‐way analysis of variance was used for comparisons among multiple groups, followed by Tukey's multiple comparisons test. *p < 0.05 versus the control group. ^#^ *p* < 0.05 versus the treat + oe‐NC group.

We treated DRG neurons and macrophages with LPS (10 mg) under hypoxia (2% O_2_) and simultaneously increased the expression of PRMT8 to investigate the effects of PRMT8 overexpression on neuronal ferroptosis and macrophage polarization in SCI. First, qRT‐PCR and western blotting were performed to detect the expression of PRMT8. PRMT8 was downregulated in the treatment group (vs. the control group) and upregulated in the treatment + oe‐*PRMT8* group (vs. the treatment + oe‐NC group) (Figure 2A). The viability of the DRG neurons was determined using the CCK‐8 assay. The treatment group showed lower viability than the control group, whereas the treatment + oe‐*PRMT8* group showed higher viability than the treatment + oe‐NC group (Figure 2B). The fluorescent probe FeRhoNoxTM‐1 was used to detect the active iron content in DRG neurons, and western blotting was used to detect the ferroptosis markers 4‐HNE and HO‐1. Compared with the control group, the treatment group showed higher iron content and increased expression of 4‐HNE and HO‐1. The treatment + oe‐*PRMT8* group showed decreases in iron content and 4‐HNE and HO‐1 levels in comparison with the treatment + oe‐NC group (Figure 2C,D). Flow cytometry was used to determine the ratio of M2 to M1 macrophages. The treatment group had a lower M2/M1 ratio than the control group, indicating that the proportion of M1 macrophages increased after LPS and hypoxia treatment. The treatment + oe‐*PRMT8* group had a higher M2/M1 ratio than the treatment + oe‐NC group, indicating that the proportion of M2 macrophages increased after overexpression of PRMT8 (Figure 2E). Immunofluorescence and western blot analyses revealed that iNOS was upregulated and Arg1 was downregulated in the treatment group (vs. the control group), which was reversed in the treatment + oe‐*PRMT8* group (vs. the treatment + oe‐NC group) (Figure 2F,G). ELISA results showed that the expression levels of IL‐6, IL‐1β, and TNF‐α increased in the treatment group (vs. the control group) and declined in the treatment + oe‐*PRMT8* group (vs. the treatment + oe‐NC group) (Figure 2H). The above data suggest that overexpression of PRMT8 could protect DRG neurons and regulate RMa‐bm polarization in vitro.

### 3.3 Overexpression of PRMT8 reduces SCI by inhibiting ferroptosis and M1 macrophage polarization

**FIGURE 3:**
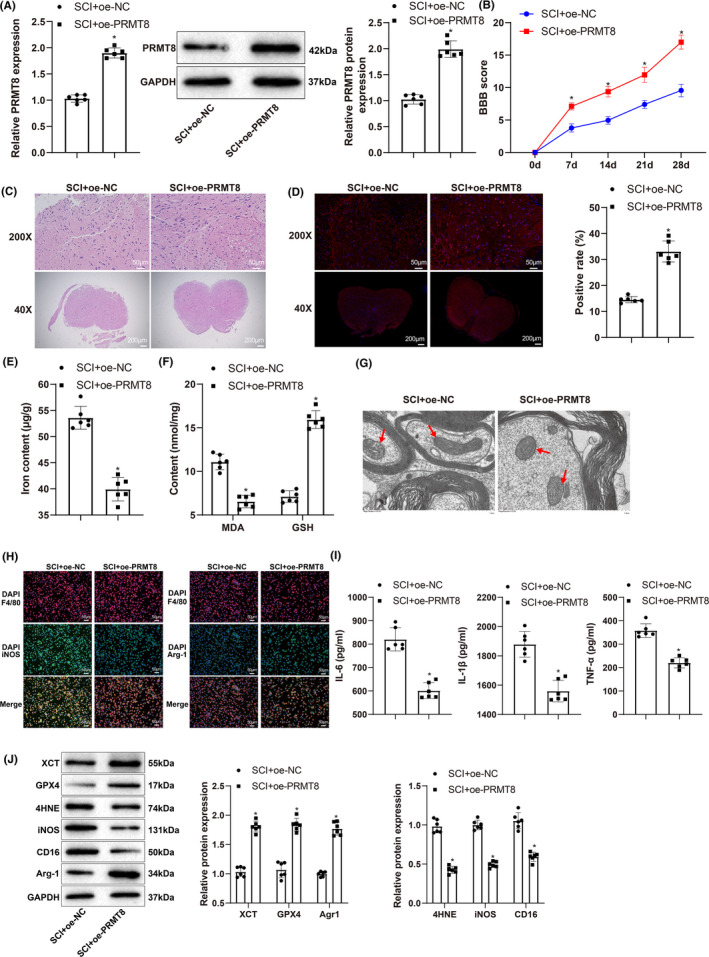
Overexpression of PRMT8 reduces SCI by inhibiting ferroptosis and M1 macrophage polarization. (A) qRT‐PCR and western blot for detecting the expression levels of PRMT8 mRNA and protein. (B) BBB score for assessing locomotion. (C) H&E staining for histopathology examination of spinal cord. (D) Immunofluorescence for revealing NeuN expression in spinal cord. (E) Iron content in spinal cord. (F) Expression levels of MDA and GSH. (G) Transmission electron microscopy for examining the morphology of mitochondria in spinal cord. (H) Immunofluorescence for revealing iNOS and Arg1 expression in spinal cord. (I) ELISA for detecting the expression of IL‐6, IL‐1β and TNF‐α. (J) Western blot for detecting the expression of XCT, GPX4, 4‐HNE, iNOS, CD16, and Agr1. The data were expressed as mean ± standard deviation. Independent samples *t*‐test was used for comparisons between two groups. The BBB scores were compared by two‐way analysis of variance. *n* = 6. *p < 0.05 versus the SCI + oe‐NC group.

To validate the function of PRMT8 in SCI, we elevated the expression of PRMT8 in rats with SCI (Figure 3A). BBB score was used to assess locomotion. The SCI + oe‐*PRMT8* group had a higher BBB score than the SCI + oe‐NC group (Figure 3B). Examination of the spinal cord by H&E staining showed that the SCI + oe‐*PRMT8* group had fewer histopathological changes than the SCI + oe‐NC group (Figure 3C). The expression of the neuronal marker NeuN, as revealed by immunofluorescence, was increased in the spinal cord of the SCI + oe‐*PRMT8* group (Figure 3D). These results indicate that overexpression of PRMT8 could reduce SCI.

Next, we investigated the effects of PRMT8 overexpression on iron overload and macrophage polarization in SCI. Using detection kits, we found that compared to the SCI + oe‐NC group, the SCI + oe‐*PRMT8* group had a lower iron content in the spinal cord (Figure 3E), a lower level of MDA, and a higher level of GSH (Figure 3F). Morphological examination of the mitochondria by TEM showed a significant reduction in mitochondrial shrinkage, mitochondrial membrane thickening, and mitochondrial cristae loss in the SCI + oe‐*PRMT8* group (Figure 3G). The images of immunofluorescence‐stained spinal cord samples showed that the expression of the M1 macrophage marker iNOS decreased, and that of the M2 macrophage marker Arg1 increased in the SCI + oe‐*PRMT8* group (Figure 3H). ELISA results showed that the SCI + oe‐*PRMT8* group had lower expression levels of IL‐6, IL‐1β, and TNF‐α than the SCI + oe‐NC group (Figure 3I). Western blot analysis revealed that the expression levels of XCT, GPX4, and Arg1 increased, while those of 4‐HNE, iNOS, and CD16 decreased in the SCI + oe‐*PRMT8* group (Figure 3J). Compared to the SCI + oe‐NC group, the SCI + oe‐*PRMT8* group also showed higher expression levels of GAP43 and NF200 (Figure S1B). These results indicate that PRMT8 overexpression could inhibit ferroptosis and M1 macrophage polarization in SCI.

### 3.4 PRMT8 catalyzes histone H3K4 methylation to activate GDNF transcription

**FIGURE 4:**
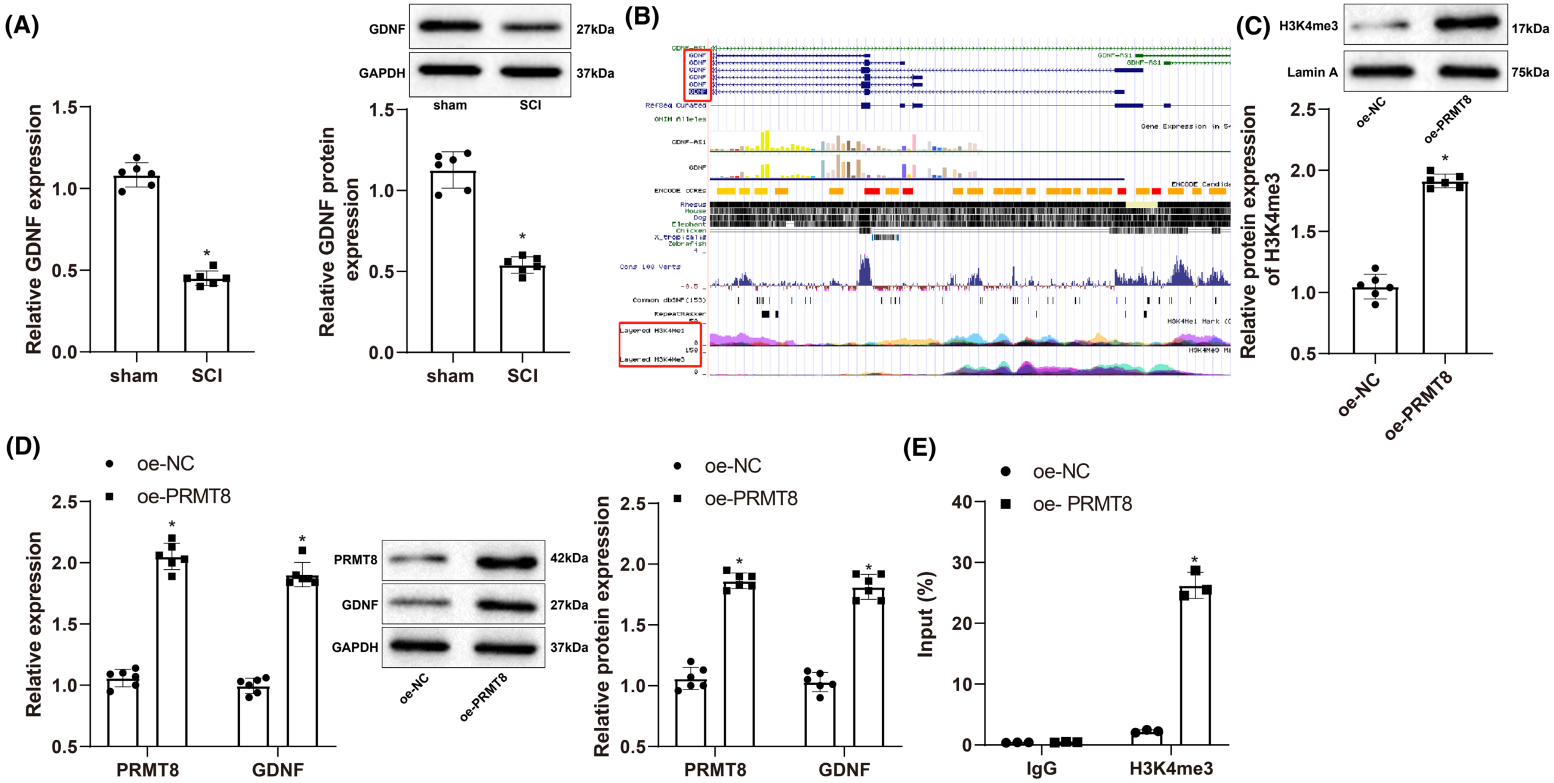
PRMT8 catalyzes histone H3K4 methylation to activate GDNF transcription. (A) qRT‐PCR and western blot for detecting the expression of GDNF mRNA and protein in SCI (*n* = 6). (B) Histone H3 methylation peaks in the promoter of GDNF gene shown by the UCSC database. (C) Western blot for detecting the expression of H3K4me3. (D) qRT‐PCR and western blot for detecting the expression of PRMT8 and GDNF. (E) ChIP for detecting the enrichment of H3K4me3 in GDNF promoter. The data were expressed as mean ± standard deviation. Independent samples *t*‐test was used for comparisons between two groups. The cell experiments were repeated thrice. *p < 0.05 versus the sham or oe‐NC group.

qRT‐PCR and western blotting revealed GDNF was expressed at low levels in SCI rats (Figure 4A). The UCSC database revealed the presence of histone H3 methylation peaks in the *GDNF* promoter (Figure 4B). To determine whether PRMT8 affects the expression of GDNF by catalyzing H3K4 methylation, we used qRT‐PCR and/or western blotting to detect the expression levels of H3K4me3, PRMT8, and GDNF. Compared to oe‐NC transfection, oe‐*PRMT8* transfection increased the expression levels of H3K4me3 (Figure 4C), PRMT8, and GDNF (Figure 4D). In the chromatin immunoprecipitation assay, H3K4me3 was significantly enriched in the *GDNF* promoter in cells transfected with oe‐*PRMT8* (Figure 4E). These data indicate that PRMT8 overexpression increases GDNF expression by activating GDNF transcription through H3K4 methylation.

### 3.5 PRMT8 overexpression regulates DRG neuron and RMa‐bm macrophage function in vitro by promoting GDNF expression

**FIGURE 5:**
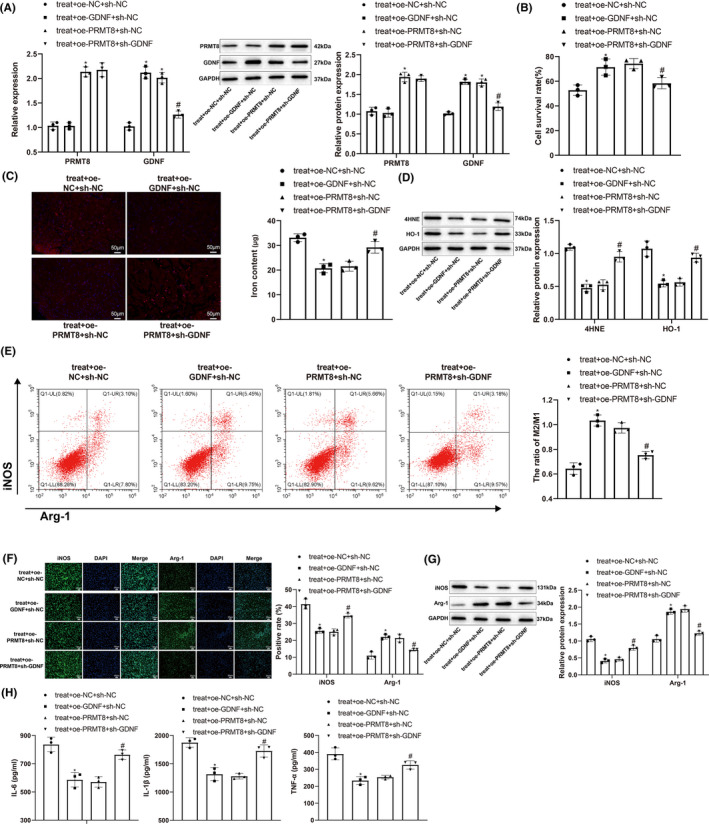
Overexpression of PRMT8 exerts therapeutic effects on DRG neurons and RMa‐bm in vitro by promoting GDNF expression. (A) qRT‐PCR and western blot for detecting the mRNA and protein expression levels of PRMT8 and GDNF. (B) CCK‐8 for detecting the survival of DRG neurons. (C) Specific fluorescent probe FeRhoNoxTM‐1 for detecting the content of active iron in DRG neurons. (D) Western blot for detecting the expression levels of 4‐HNE and HO‐1 in DRG neurons. (E) Flow cytometry for detecting M1 and M2 macrophages. (F) Immunofluorescence for visualizing the expression of iNOS and Arg1 in macrophages. (G) Western blot for detecting the expression levels of iNOS and Arg1 in macrophages. (H) ELISA for measuring the expression levels of IL‐6, IL‐1β and TNF‐α in macrophages. The data were expressed as mean ± standard deviation. One‐way analysis of variance was used for comparisons among multiple groups, followed by Tukey's multiple comparisons test. *p < 0.05 versus the treat + oe‐NC + sh‐NC group. ^#^ *p* < 0.05 versus the treat + oe‐PRMT8 + sh‐NC group.

We treated DRG neurons and RMa‐bm macrophages with LPS (10 mg) under hypoxia (2% O_2_); simultaneously, we altered the expression of PRMT8 and GDNF to investigate the regulation of GDNF by PRMT8 during neuronal ferroptosis and macrophage polarization. PRMT8 was upregulated in the treatment + oe‐*PRMT8* + sh‐NC group (vs. the treatment + oe‐NC + sh‐NC group). GDNF was upregulated in the treatment + oe‐*GDNF* + sh‐NC group (vs. the treatment + oe‐NC + sh‐NC group) and downregulated in the treatment + oe‐*PRMT8* + sh‐*GDNF* group (vs. the treatment + oe‐*PRMT8* + sh‐NC group) (Figure 5A). For DRG neurons, CCK‐8, fluorescent probe FeRhoNoxTM‐1, and western blotting were used to detect cell viability, active iron content, and ferroptosis markers, respectively. Compared with the treatment + oe‐NC + sh‐NC group, the treatment + oe‐*PRMT8* + sh‐NC and treatment + oe‐*GDNF* + sh‐NC groups showed higher survival rates, lower iron content, and decreased levels of 4‐HNE and HO‐1. The treatment + oe‐*PRMT8* + sh‐*GDNF* group showed a decrease in viability and increases in iron content and 4‐HNE and HO‐1 levels in comparison with the treatment + oe‐*PRMT8* + sh‐NC group (Figure 5B–D). Flow cytometry was used to determine the ratio of M2 to M1 macrophages. The treatment + oe‐*PRMT8* + sh‐NC and treatment + oe‐*GDNF* + sh‐NC groups had higher M2/M1 ratios than the treatment + oe‐NC + sh‐NC group, indicating that the proportion of M2 macrophages increased after overexpression of PRMT8 or GDNF. The treatment + oe‐*PRMT8* + sh‐*GDNF* group had a lower M2/M1 ratio than the treatment + oe‐*PRMT8* + sh‐NC group, indicating that sh‐*GDNF* increased the proportion of M1 macrophages in the presence of oe‐*PRMT8* (Figure 5E). Immunofluorescence and western blot analyses revealed that iNOS was downregulated and Arg1 was upregulated in the treatment + oe‐*PRMT8* + sh‐NC and treatment + oe‐*GDNF* + sh‐NC groups (vs. the treatment + oe‐NC + sh‐NC group), which was reversed in the treatment + oe‐*PRMT8* + sh‐*GDNF* group (vs. the treatment + oe‐*PRMT8* + sh‐NC group) (Figure 5F,G). The ELISA results showed that the expression levels of IL‐6, IL‐1β, and TNF‐α declined in the treatment + oe‐*PRMT8* + sh‐NC and treatment + oe‐*GDNF* + sh‐NC groups (vs. the treatment + oe‐NC + sh‐NC group) and increased in the treatment + oe‐*PRMT8* + sh‐*GDNF* group (vs. the treatment + oe‐*PRMT8* + sh‐NC group) (Figure 5H). The above data suggest that overexpression of PRMT8 regulates DRG neurons and RMa‐bm function in vitro by promoting GDNF expression.

### 3.6 Overexpression of PRMT8 reduces SCI by increasing GDNF expression

**FIGURE 6:**
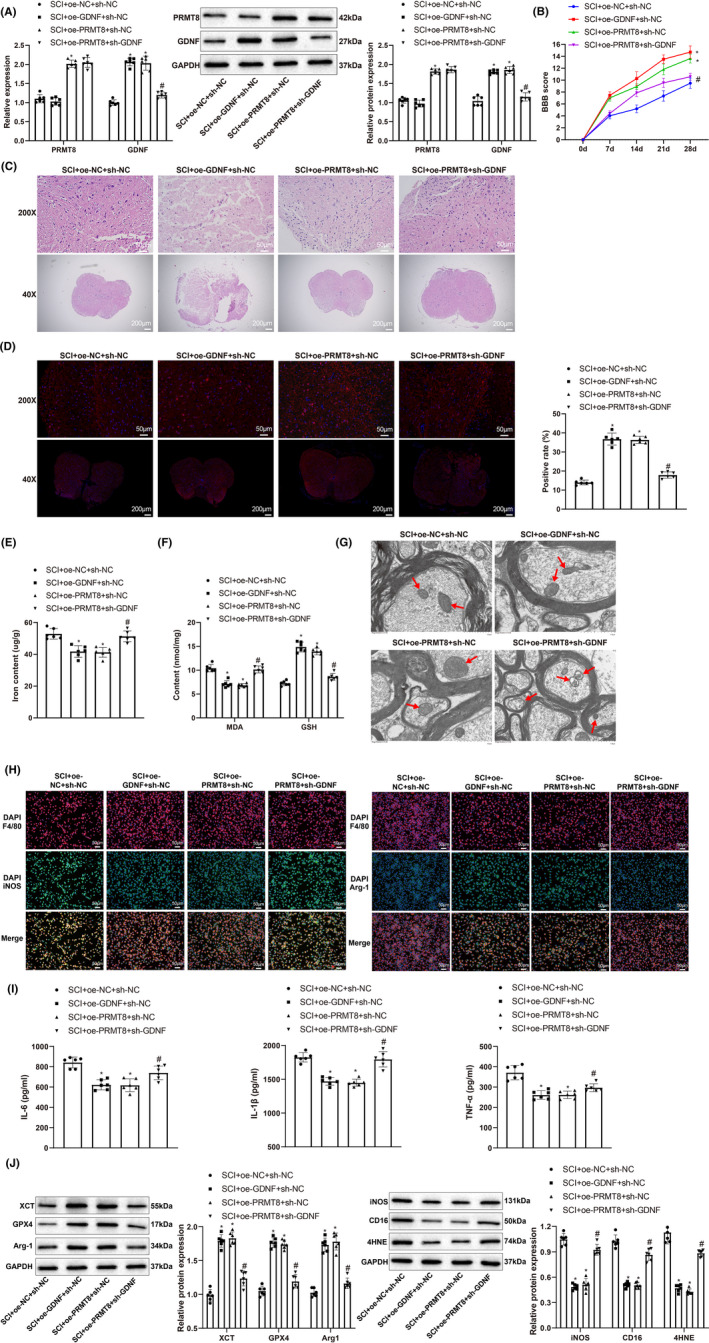
Overexpression of PRMT8 reduces SCI by increasing GDNF expression. (A) qRT‐PCR and western blot for detecting the mRNA and protein expression levels of PRMT8 and GDNF. (B) BBB score for assessing locomotion. (C) H&E staining for histopathology examination of spinal cord. (D) Immunofluorescence for revealing NeuN expression in spinal cord. (E) Iron content in spinal cord. (F) Expression levels of MDA and GSH. (G) Transmission electron microscopy for examining the morphology of mitochondria in spinal cord. (H) Immunofluorescence for revealing iNOS and Arg1 expression in spinal cord. (I) ELISA for detecting the expression of IL‐6, IL‐1β and TNF‐α. (J) Western blot for detecting the expression of XCT, GPX4, 4‐HNE, iNOS, CD16, and Agr1. The data were expressed as mean ± standard deviation. One‐way analysis of variance was used for comparisons among multiple groups, followed by Tukey's multiple comparisons test. The BBB scores were compared by two‐way analysis of variance. *n* = 6. *p < 0.05 versus the SCI + oe‐NC + sh‐NC group. ^#^ *p* < 0.05 versus the SCI + oe‐PRMT8 + sh‐NC group.

To confirm the regulation of PRMT8 on GDNF in SCI, we altered the expression of PRMT8 and GDNF in rats with SCI. qRT‐PCR and western blotting showed that PRMT8 was upregulated in the SCI + oe‐*PRMT8* + sh‐NC group (vs. the SCI + oe‐NC + sh‐NC group). GDNF was upregulated in the SCI + oe‐*GDNF* + sh‐NC group (vs. the SCI + oe‐NC + sh‐NC group) and downregulated in the SCI + oe‐*PRMT8* + sh‐*GDNF* group (vs. the SCI + oe‐*PRMT8* + sh‐NC group) (Figure 6A). The BBB score, H&E staining, and immunofluorescence were used to detect changes in locomotion, spinal cord histopathology, and NeuN expression, respectively. Compared to the SCI + oe‐NC + sh‐NC group, the SCI + oe‐*PRMT8* + sh‐NC and SCI + oe‐*GDNF* + sh‐NC groups showed higher BBB scores, less tissue damage, and increased NeuN expression. The SCI + oe‐*PRMT8* + sh‐*GDNF* group showed reductions in BBB score and NeuN expression and increases in tissue damage and cavity area compared to the SCI + oe‐*PRMT8* + sh‐NC group (Figure 6B–D). To investigate the influence of PRMT8/GDNF on ferroptosis, we measured the iron content and MDA and GSH levels in the spinal cord using detection kits and observed the morphology of the mitochondria in the spinal cord using transmission electron microscopy. Compared to the SCI + oe‐NC + sh‐NC group, the SCI + oe‐*PRMT8* + sh‐NC and SCI + oe‐*GDNF* + sh‐NC groups had lower iron content and MDA levels, higher GSH levels, and less mitochondrial damage. There were significant increases in iron content, MDA level, and mitochondrial damage, and a decrease in GSH level in the SCI + oe‐*PRMT8* + sh‐*GDNF* group compared with the SCI + oe‐*PRMT8* + sh‐NC group (Figure 6E–G). Immunofluorescence and western blot analyses revealed that the expression levels of XCT, GPX4, and Arg1 were increased in the SCI + oe‐*PRMT8* + sh‐NC and SCI + oe‐*GDNF* + sh‐NC groups (vs. the SCI + oe‐NC + sh‐NC group) and decreased in the SCI + oe‐*PRMT8* + sh‐*GDNF* group (vs. the SCI + oe‐*PRMT8* + sh‐NC group). In contrast, the expression levels of 4‐HNE, iNOS, and CD16 were decreased in the SCI + oe‐*PRMT8* + sh‐NC and SCI + oe‐*GDNF* + sh‐NC groups (vs. the SCI + oe‐NC + sh‐NC group) and increased in the SCI + oe‐*PRMT8* + sh‐*GDNF* group (vs. the SCI + oe‐*PRMT8* + sh‐NC group) (Figure 6H,J). The ELISA results showed that the expression levels of IL‐6, IL‐1β and TNF‐α were reduced in the SCI + oe‐*PRMT8* + sh‐NC and SCI + oe‐*GDNF* + sh‐NC groups (vs. the SCI + oe‐NC + sh‐NC group) and elevated in the SCI + oe‐*PRMT8* + sh‐*GDNF* group (vs. the SCI + oe‐*PRMT8* + sh‐NC group) (Figure 6I). Moreover, the western blot analysis revealed that the expression levels of neurogenesis‐related proteins GAP43 and NF200 were elevated in the SCI + oe‐*PRMT8* + sh‐NC and SCI + oe‐*GDNF* + sh‐NC groups (vs. the SCI + oe‐NC + sh‐NC group) and reduced in the SCI + oe‐*PRMT8* + sh‐*GDNF* group (vs. the SCI + oe‐*PRMT8* + sh‐NC group) (Figure S1C). Altogether, these results indicate that PRMT8 overexpression may inhibit ferroptosis and M1 macrophage polarization by increasing GDNF expression, thereby alleviating SCI.

## 4 DISCUSSION

The pathophysiology of SCI comprises an initial traumatic insult and progressive secondary injury characterized by ischemia, excitotoxicity, pro‐apoptotic signaling, and peripheral inflammatory cell infiltration.^19^ Better understanding the heterogeneity of SCI has translated to the novel management of SCI, which specifically targets SCI subpopulations using biomarkers.^20^ This study revealed low PRMT8 expression in SCI, which contributed to neuronal ferroptosis and M1 macrophage polarization. As an arginine methyltransferase, PRMT8 catalyzes H3K4 methylation of the *GDNF* promoter and activates GDNF transcription. Upregulation of PRMT8/GDNF can improve locomotion and reduce tissue damage after SCI by inhibiting neuronal ferroptosis and M1 macrophage polarization.

The induction of ferroptosis depends on two essential factors: a substantial labile iron pool and inactivation of antioxidative ability. Iron overload induces oxidative injury mainly by catalyzing the Fenton reaction, during which relatively harmless ROS are transformed into highly reactive hydroxyl radicals that can attack biological macromolecules when cellular antioxidant defenses are overwhelmed.^21^ The non‐canonical ferroptotic pathway is triggered by an increase in intracellular labile Fe^2+^ upon excessive activation of HO‐1.^22^ GSH is a critical substrate of GPX4 that reduces ROS, and its synthesis requires three materials: glutamine, glycine, and cystine.^23^ Cystine is imported into cells by XCT, which is downregulated during ferroptosis.^24^ Peroxidation of polyunsaturated fatty acids in membrane phospholipids following the accumulation of ROS is an important process in ferroptosis, which produces initial lipid hydroperoxides and subsequent reactive aldehydes such as MDA and 4‐HNE.^25^, ^26^ Subsequently, the membrane structure is destroyed, and cell death occurs. In this study, overexpression of PRMT8 increased the viability of DRG neurons after treatment with LPS under hypoxic conditions and decreased the iron content and 4‐HNE and HO‐1 levels in these cells. In rat models of SCI, overexpression of PRMT8 reduced iron content, mitochondrial damage, and the production of MDA and 4‐HNE, while increasing the expression of GSH, XCT, and GPX4. These data indicate that PRMT8 protects neurons against ferroptosis in SCI.

Macrophages are of monocyte origin and are distributed in various body tissues where they predominantly regulate innate and adaptive immunity.^27^ Macrophages can be categorized into two subtypes according to their function: classically activated M1 and alternatively activated M2. Macrophages obtain M1 phenotypes in toll‐like receptor‐ and interferon‐dominated inflammatory settings, and they produce various proinflammatory cytokines, while M2 polarization is driven by Th2 responses and is characterized by high levels of IL‐10 and Arg1.^28^ In addition to ferroptosis, PRMT8 also apparently regulates macrophage polarization in SCI. PRMT8 overexpression reduced M1 macrophages, inhibited iNOS and CD16 expression, and increased Arg1 expression in SCI models. Moreover, PRMT8 overexpression suppressed the expression of IL‐6, IL‐1β, and TNF‐α.

PRMT8 plays a specific role in neuronal functional events in the somatosensory, limbic, and motor systems.^29^ Given its role, dysregulation of PRMT8 may be implicated in motoneuron‐related degenerative diseases (such as amyotrophic lateral sclerosis) and neurodevelopmental disorders.^30^ Knockout of *PRMT8* induces progressive muscle atrophy and neuromuscular junction breakage in aging mice by disrupting the repair of DNA double‐stranded breaks in spinal cord motoneurons by blocking the CREB1‐dependent transcriptional activation of neuroprotective genes.^31^ Mice with deletion of *PRMT8* in the brain showed synaptic function and plasticity defects in the hippocampus and impaired fear learning.^32^ PRMT8 strengthens the actin cytoskeleton for dendritic spine maturation by methylating G3BP1 and suppressing the subsequent activation of the Rac1/PAK1 signaling pathway.^33^ However, there is scant evidence supporting the effects of PRMT8 on ferroptosis and macrophage polarization. Therefore, we further searched for the downstream targets of PRMT8 that may be associated with these events. The experimental results indicated that PRMT8 promoted the transcriptional activation of GDNF by catalyzing H3K4 methylation.

Since GDNF benefits the survival and regeneration of dopaminergic neurons, it has been exploited in a large number of clinical trials using infusion or gene delivery methods to treat Parkinson's disease.^34^ GDNF therapy has also shown neuroprotective effects against SCI, such as neural stem cell proliferation, axonal regeneration, apoptosis inhibition, tissue reconstruction, and functional recovery.^35^, ^36^, ^37^ Zheng et al. found that long noncoding RNA (lncRNA) GDNF‐AS1, together with other ferroptosis‐related lncRNAs, could be used to form a prognostic signature to predict the overall survival and immune response of patients with glioma.^38^ Macrophage delivery of GDNF improves motor and non‐motor dysfunction, reduces dopaminergic neuron loss, and preserves axonal terminals in mouse models of Parkinson's disease.^39^ Thus, GDNF is correlated with ferroptosis and macrophage expression of GDNF can be used to restore neurological function. Our data showed that overexpression of GDNF exerted neuroprotective and anti‐inflammatory effects similar to those of GDNF overexpression in SCI models. More importantly, the knockdown of *GDNF* reversed the therapeutic effects of GDNF overexpression.

The link between macrophage polarization and ferroptosis has not been definitively concluded in existing literature. Macrophages can trigger ferroptosis through multiple signaling pathways, and ferroptosis products in turn regulate tumor‐associated macrophage polarization.^40^ For example, TGF‐β1, which can be released by macrophages, promotes tumor cell ferroptosis by activating SMAD‐mediated signaling.^41^ Dai et al. found that autophagy‐dependent ferroptosis drives M2‐like macrophage polarization in pancreatic tumors through transmission of the KRAS protein.^42^ They also found that the loss of GPX4 or a high‐iron diet stimulates ferroptosis through the 8‐OHG/TMEM173 DNA sensor pathway, which in turn increases macrophage accumulation and activation in KRAS‐driven pancreatic tumors.^43^ Ferroptosis‐conditioned medium stimulates proinflammatory cytokine production by peritoneal macrophages, and treatment with the ferroptosis inhibitor ferrostatin‐1 and the inducer RSL3, the latter of which causes ferroptosis resistance in peritoneal macrophages, suppresses neuroinflammation in LPS‐treated mice.^44^ These findings suggest that there may be an interactive relationship between macrophage polarization and ferroptosis in SCI, which was not clarified in this study. In subsequent studies, the relationship between macrophage polarization and ferroptosis in SCI can be explored by applying ferroptosis inhibitors or intervening in macrophage polarization.

In summary, PRMT8 may alleviate SCI by inhibiting neuronal ferroptosis and M1 macrophage polarization through the upregulation of GDNF. Novel therapies for SCI can target molecules involved in ferroptosis and macrophage polarization, and PRMT8 and GDNF are promising candidates. However, the downstream pathways by which PRMT8 and GDNF regulate SCI were not addressed in this study and are worthy of further investigation. Moreover, clinical trials are needed to translate PRMT8/GDNF‐based precision therapy into clinical practice. Additionally, merely mitigating secondary injury may have limited effects because of the disruption of axons at the time of injury.^45^ Therefore, strategies for axon recovery, combined with neuroprotection and anti‐inflammatory approaches, may exert greater effects in the repair of injured spinal cord.

## CONFLICT OF INTEREST STATEMENT

The authors declare there is no conflict of interest regarding this study.

## Supporting information


Figure S1

Appendix S1

## Data Availability

The data that support the findings of this study are available from the corresponding author upon reasonable request.
