# Supplementary figures and images for: Protein arginine methyltransferase 8 regulates ferroptosis and macrophage polarization in spinal cord injury via glial cell‐derived neurotrophic factor
Source: CNS Neurosci Ther. 2023 Mar 13;29(8):2145–61. doi: 10.1111/cns.14162 (PMC10352898; doi:10.1111/cns.14162)

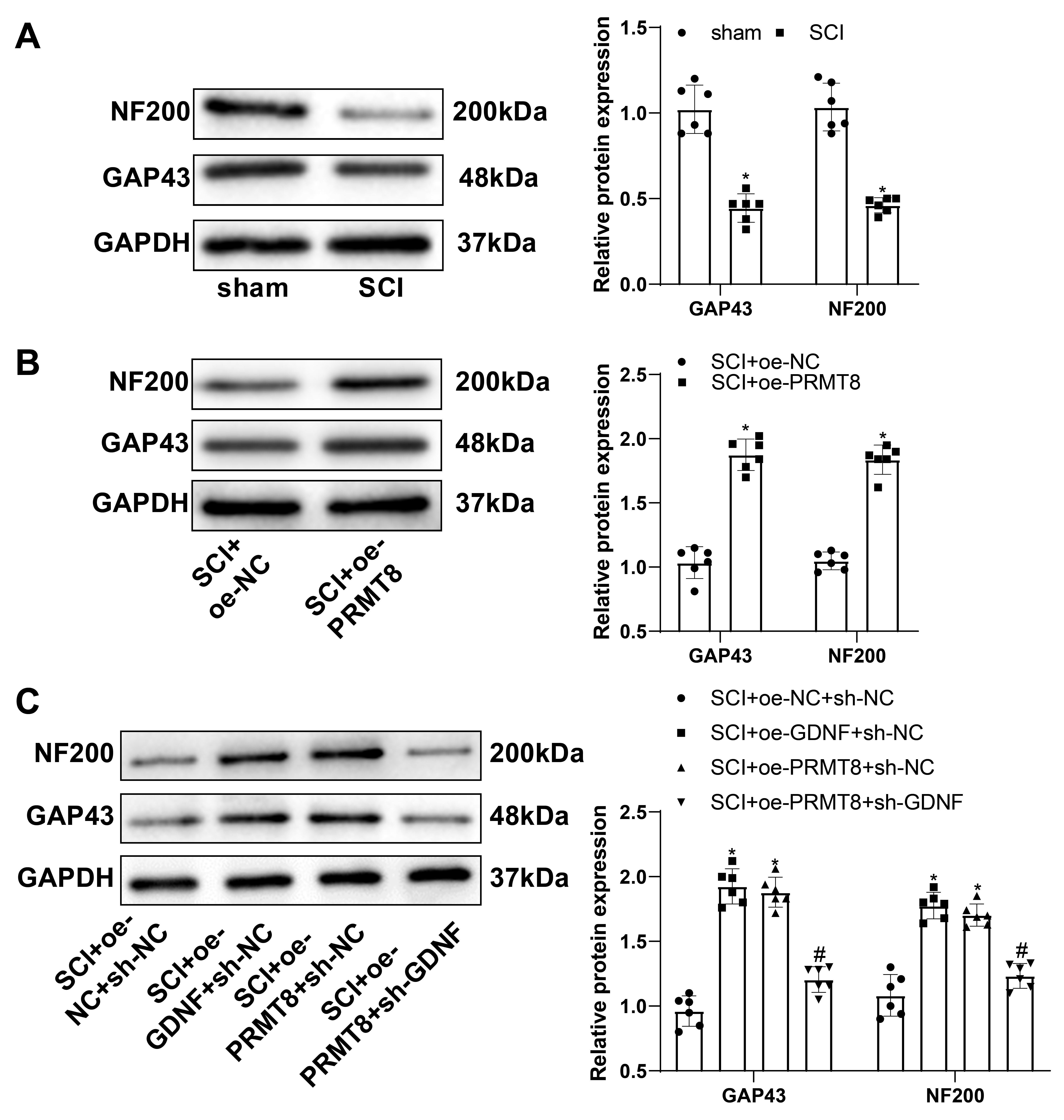

Supplement: Supplementary file 1 — Figure S1 [file CNS-29-2145-s001.jpg]
