# Supplementary material for: Protein arginine methyltransferase 8 regulates ferroptosis and macrophage polarization in spinal cord injury via glial cell‐derived neurotrophic factor
Source: CNS Neurosci Ther. 2023 Mar 13;29(8):2145–61. doi: 10.1111/cns.14162 (PMC10352898; doi:10.1111/cns.14162)

The original image of westernblot

Full unedited gel/blot for Figure 1J GAPDH

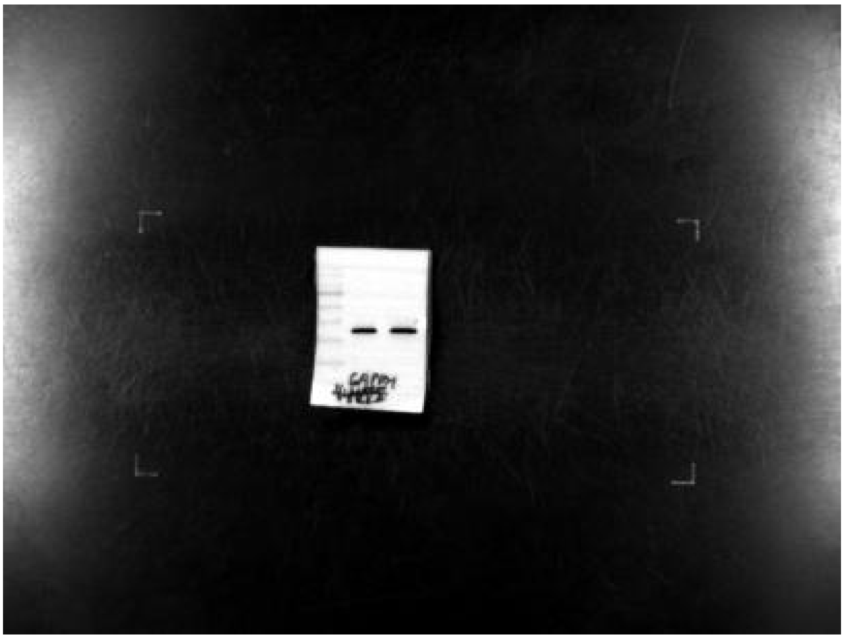

Full unedited gel/blot for Figure 1J 4HNE

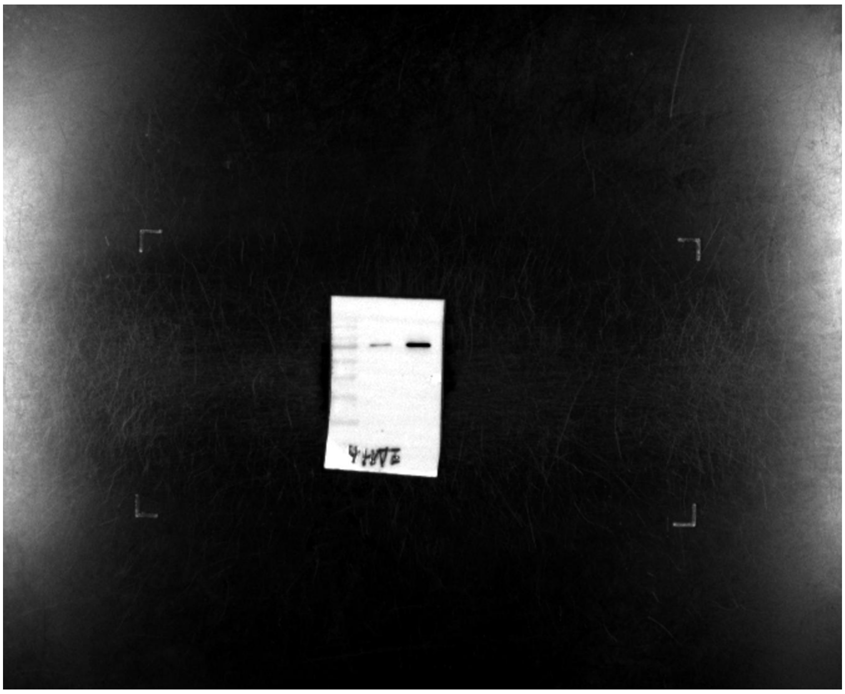

Full unedited gel/blot for Figure 1J Arg1

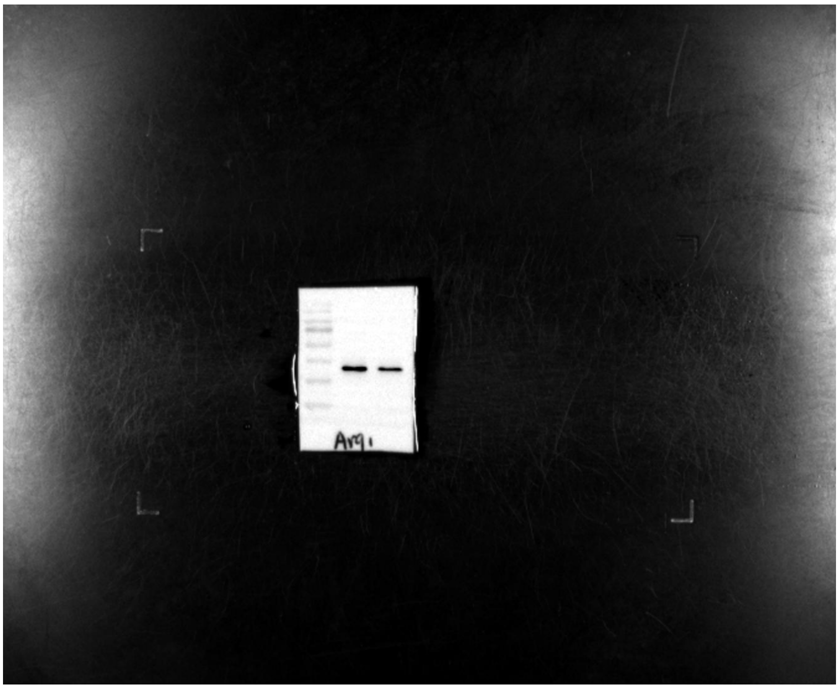

Full unedited gel/blot for Figure 1J CD16

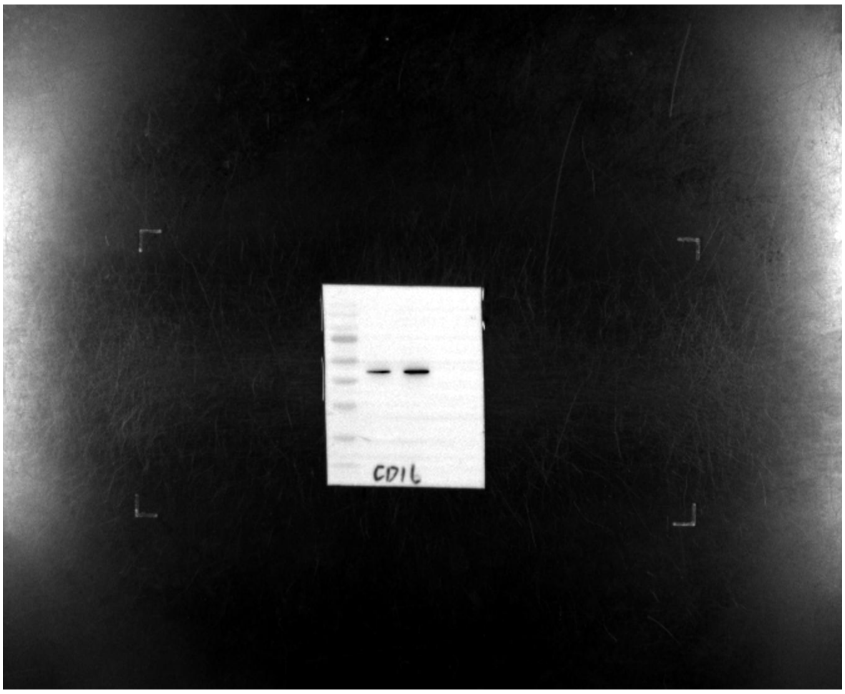

Full unedited gel/blot for Figure 1J GPX4

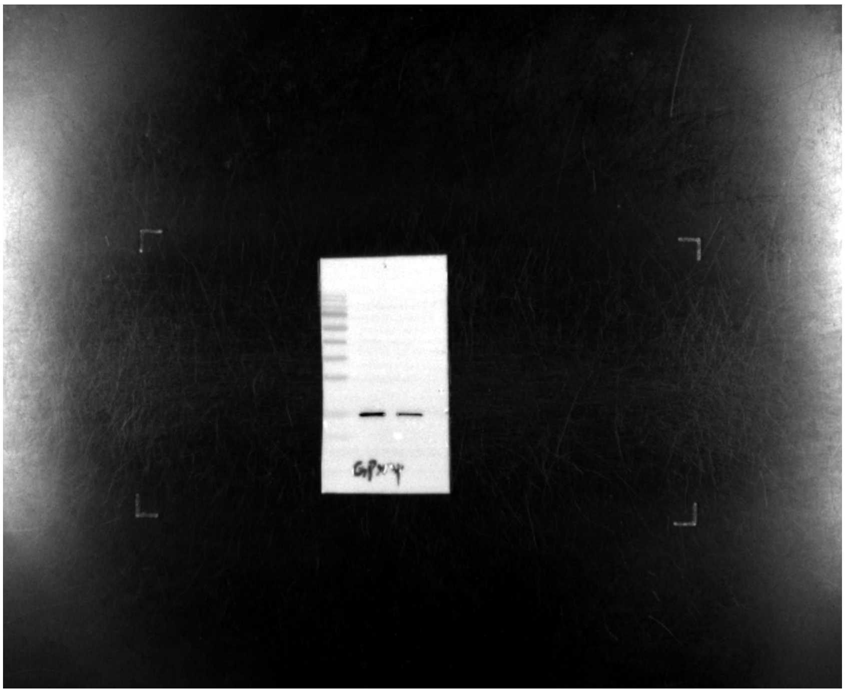

Full unedited gel/blot for Figure 1J INOS

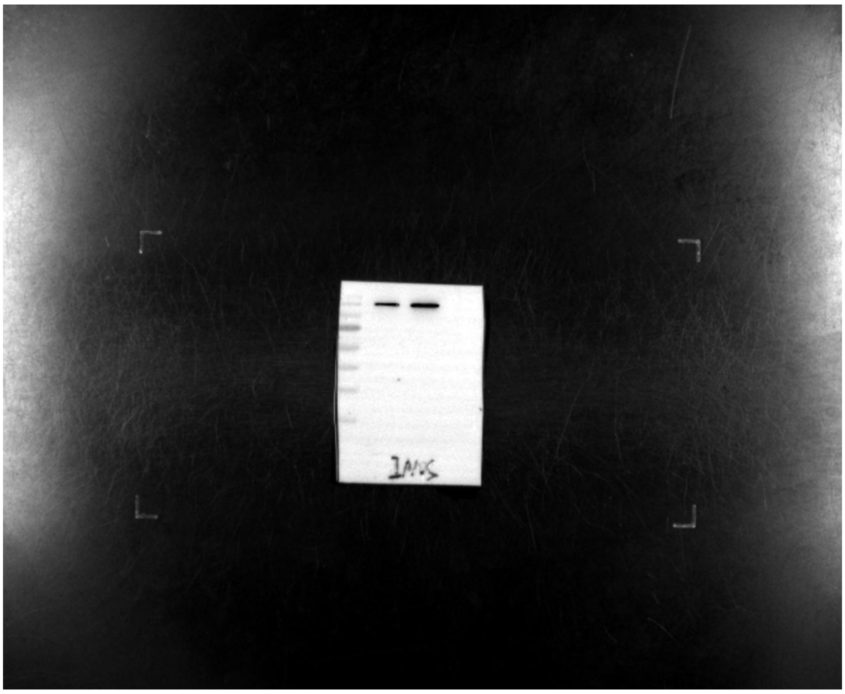

Full unedited gel/blot for Figure 1J XCT

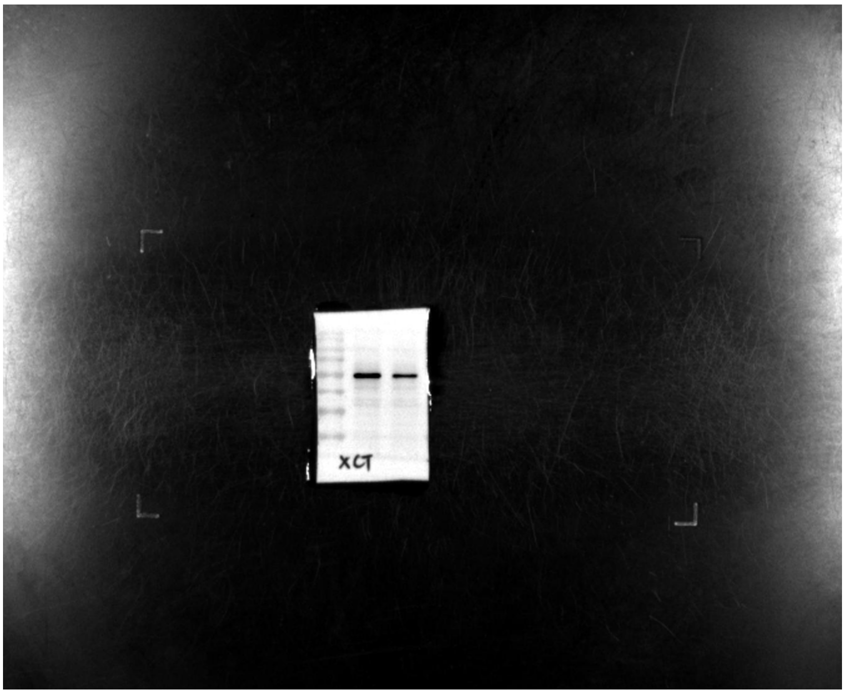

Full unedited gel/blot for Figure 1K GAPDH

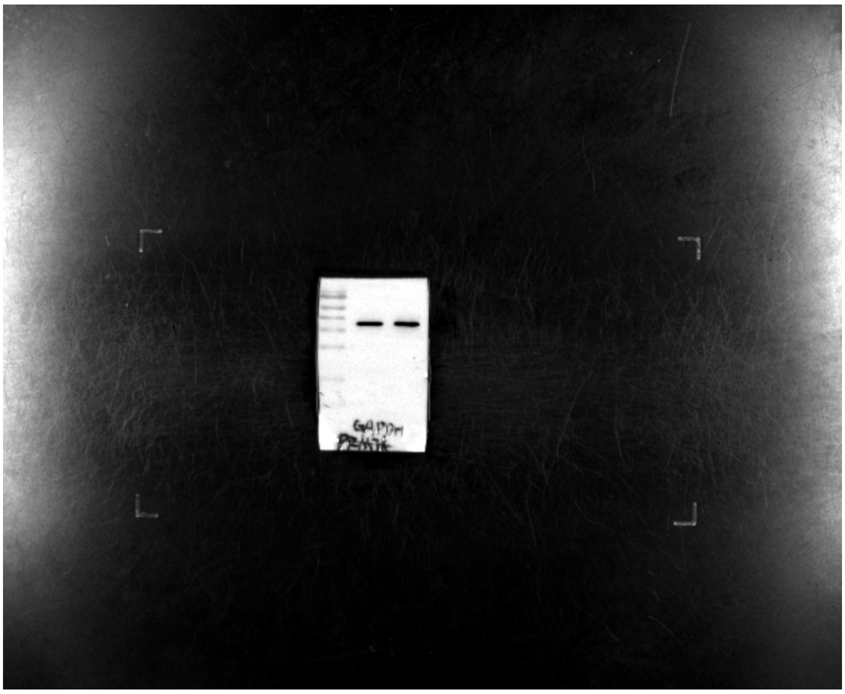

Full unedited gel/blot for Figure 1K PRMT8

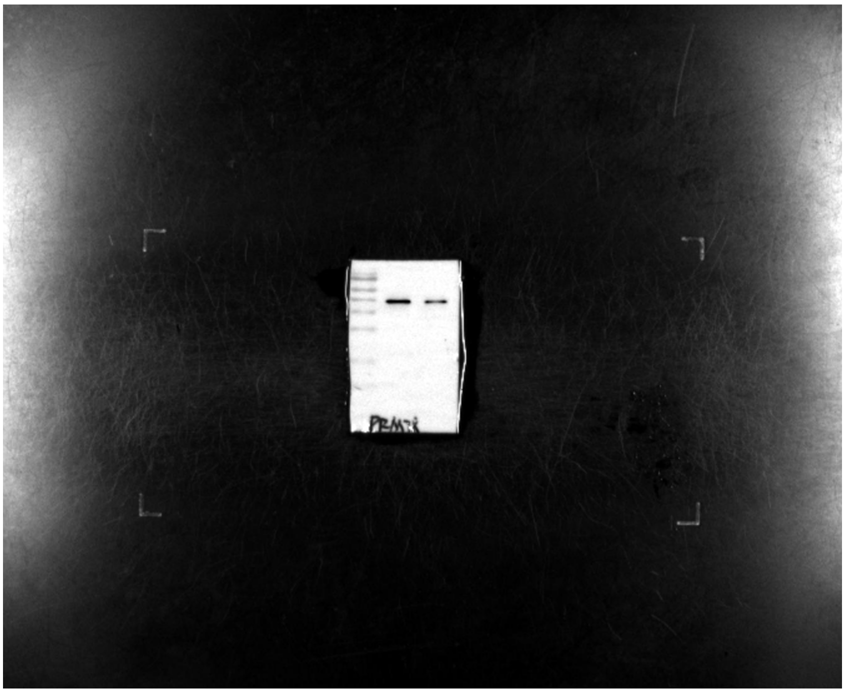

Full unedited gel/blot for Figure 2A GAPDH

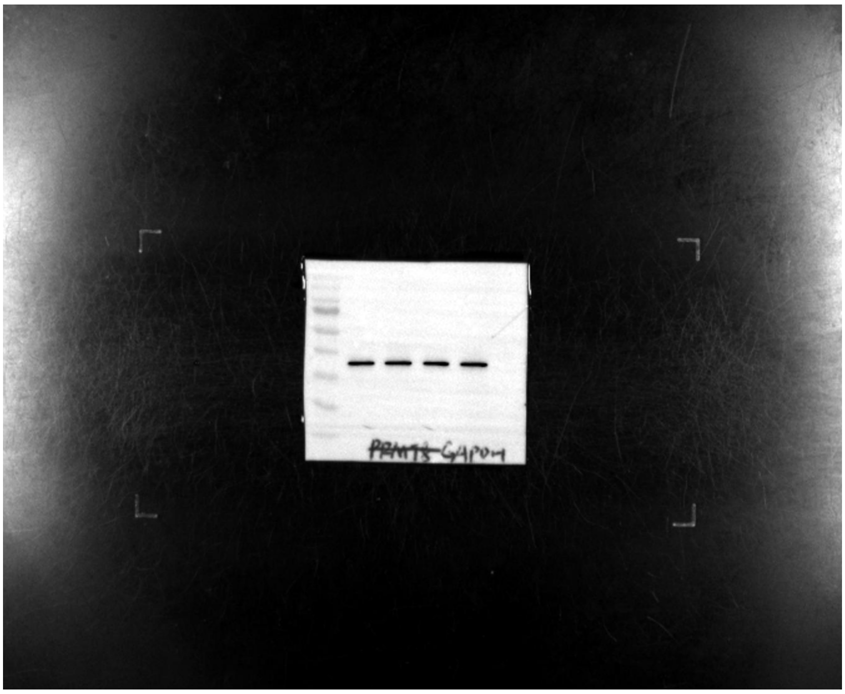

Full unedited gel/blot for Figure 2A PRMT8

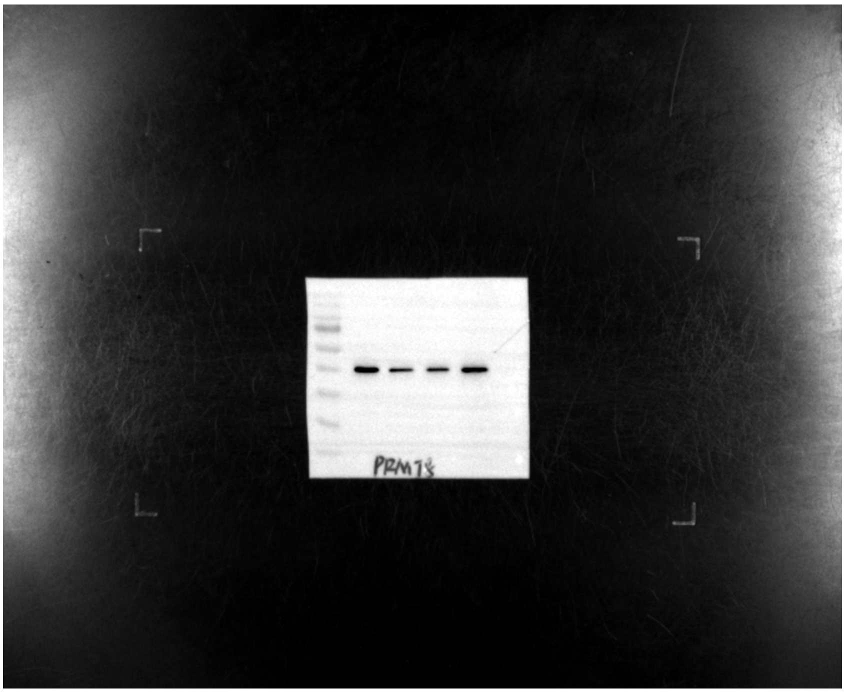

Full unedited gel/blot for Figure 2D GAPDH

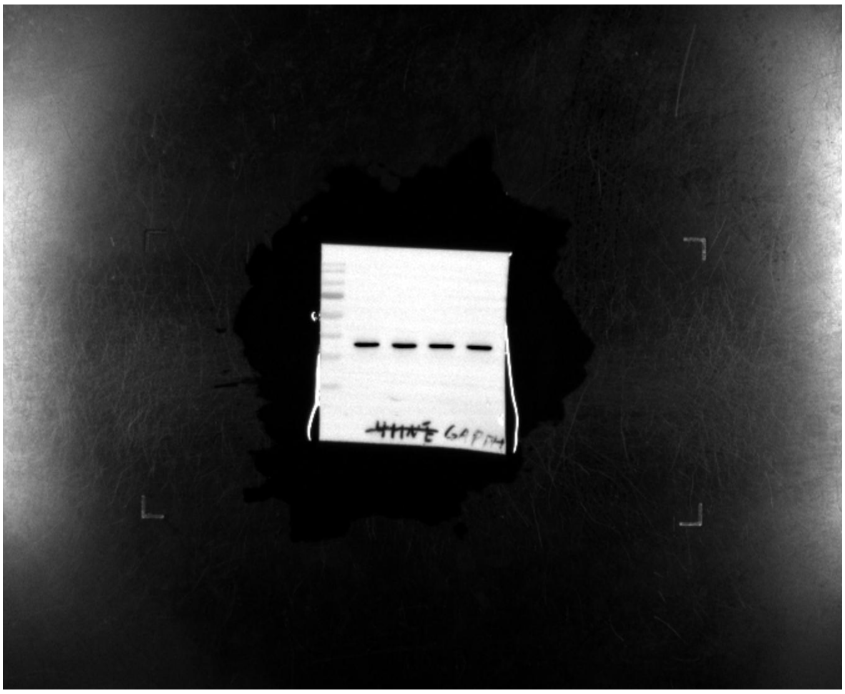

Full unedited gel/blot for Figure 2D 4HNE

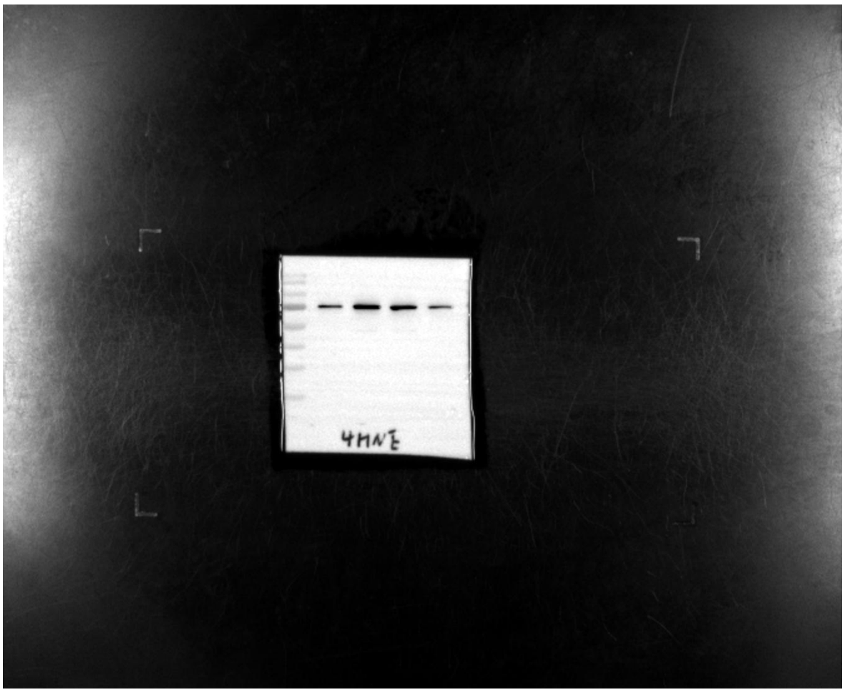

Full unedited gel/blot for Figure 2D HO-1

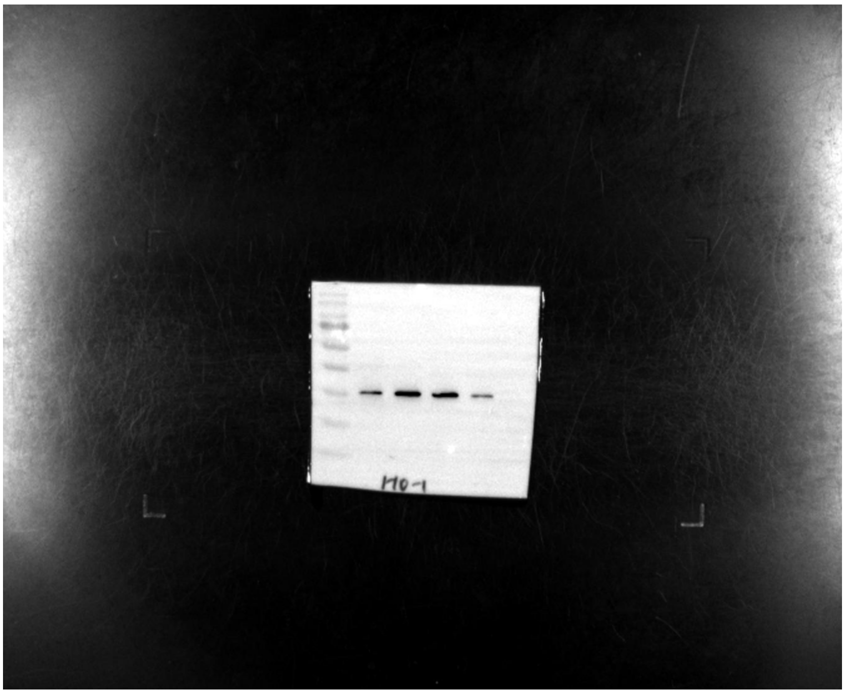

Full unedited gel/blot for Figure 2G GAPDH

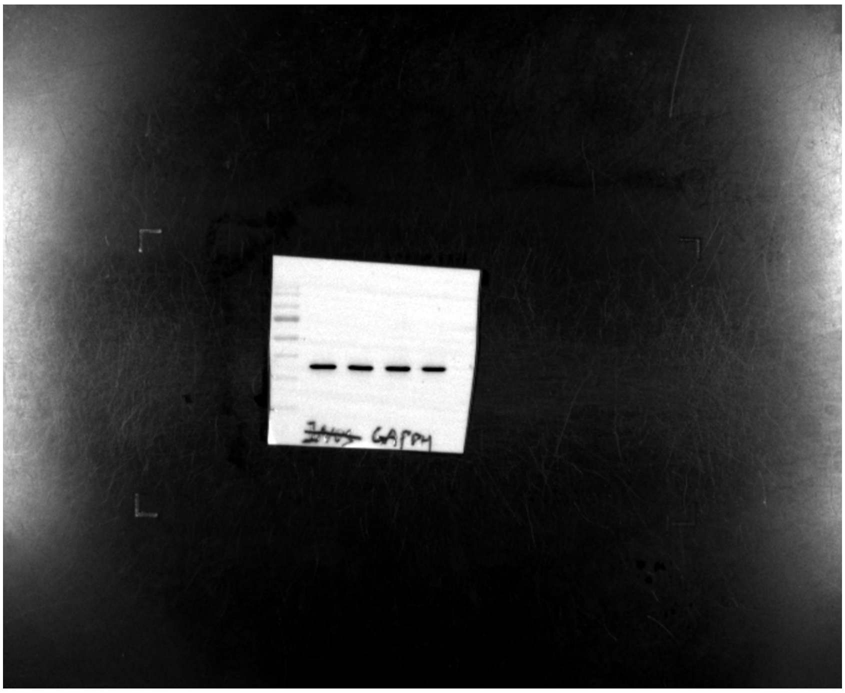

Full unedited gel/blot for Figure 2G Arg-1

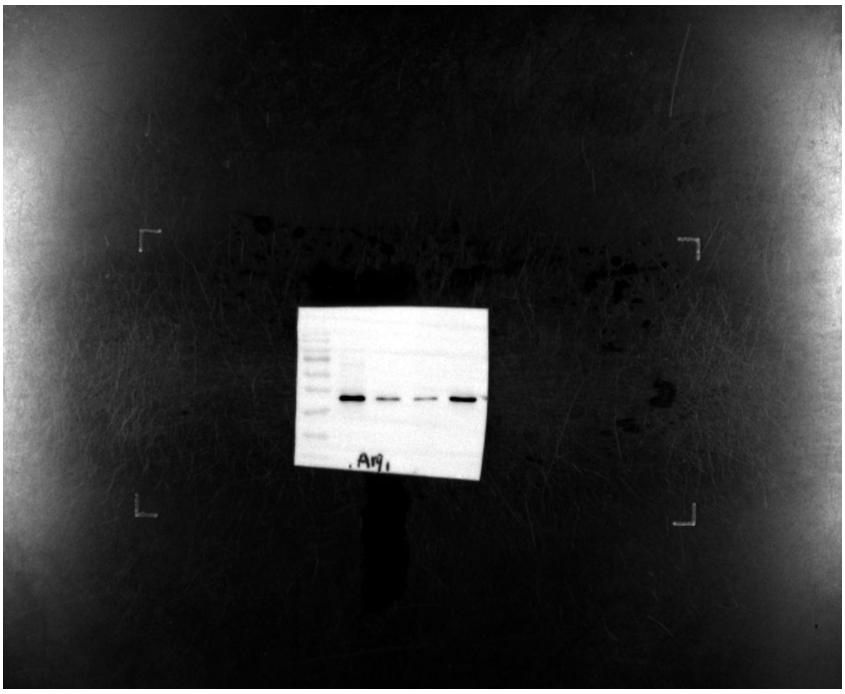

Full unedited gel/blot for Figure 2G INOS

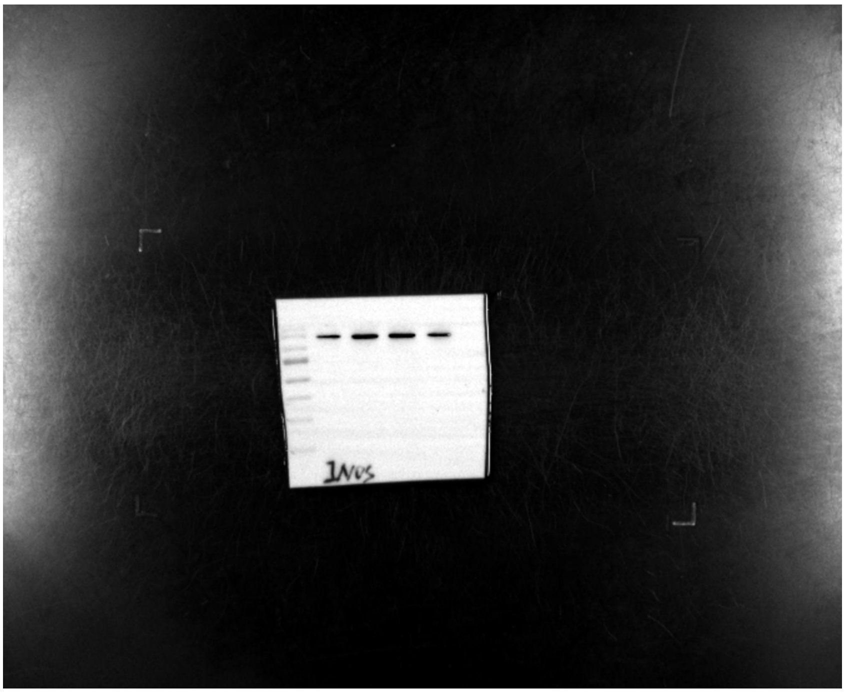

Full unedited gel/blot for Figure 3A GAPDH

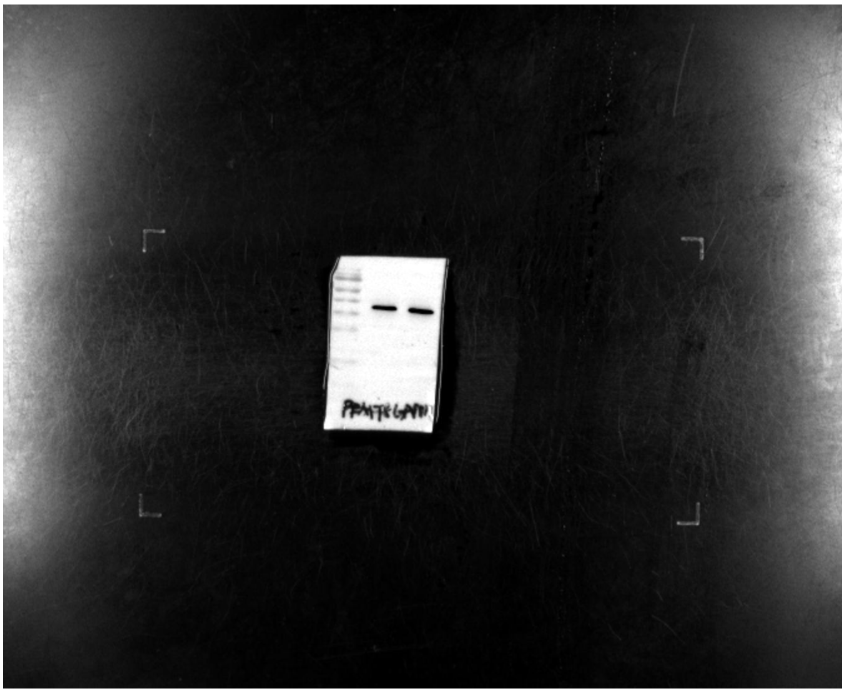

Full unedited gel/blot for Figure 3A PRMT8

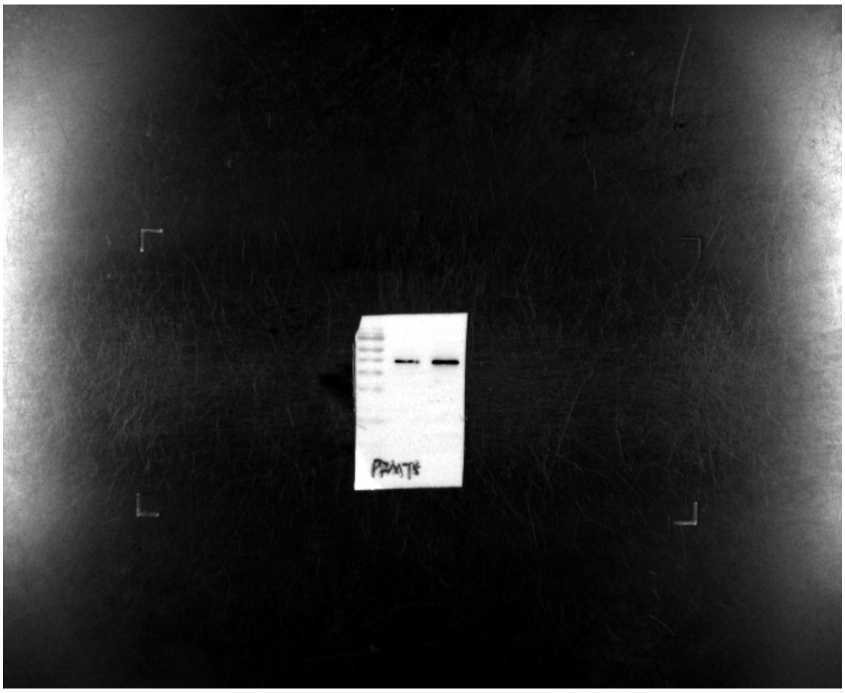

Full unedited gel/blot for Figure 3J GAPDH

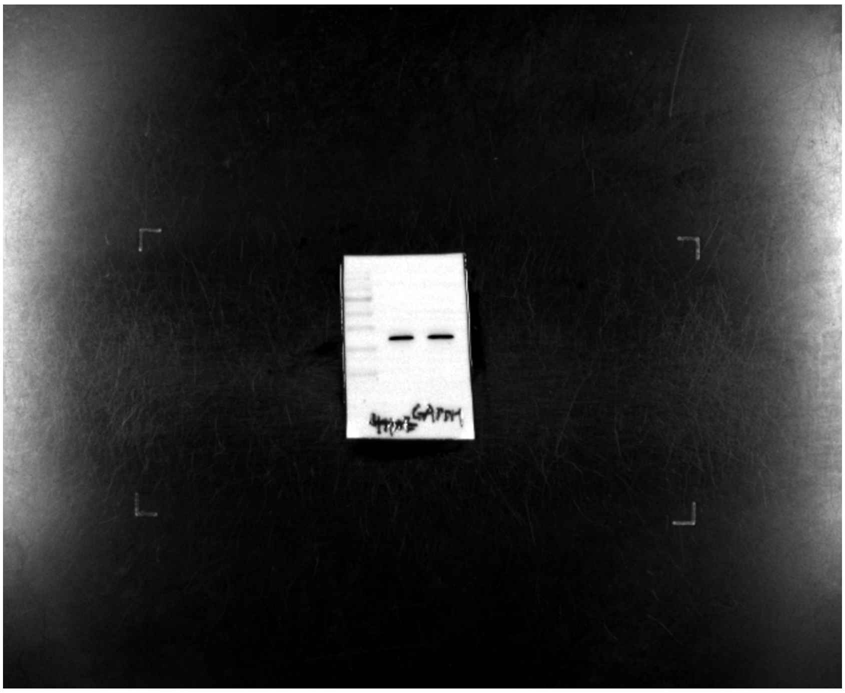

Full unedited gel/blot for Figure 3J 4HNE

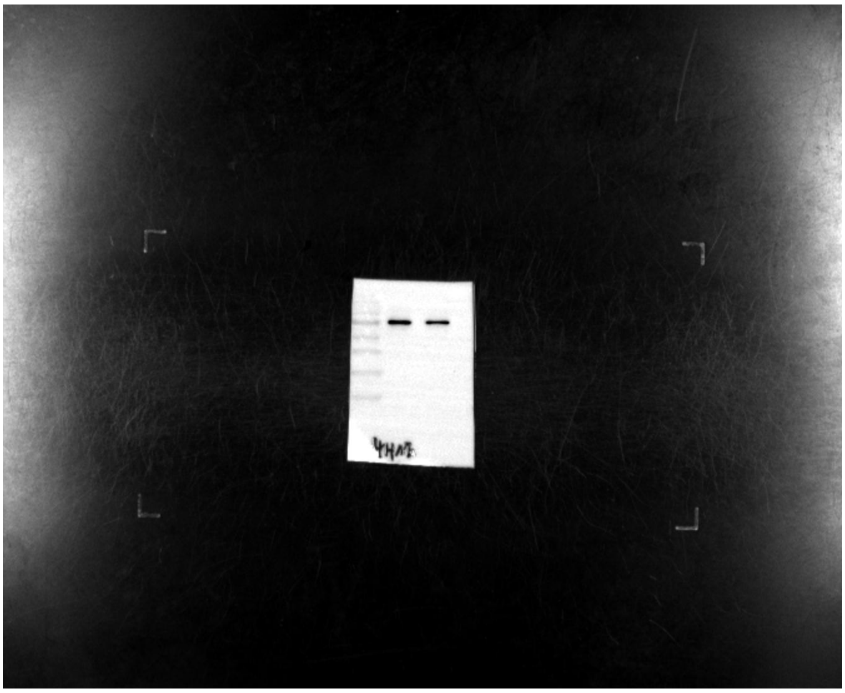

Full unedited gel/blot for Figure 3J Arg-1

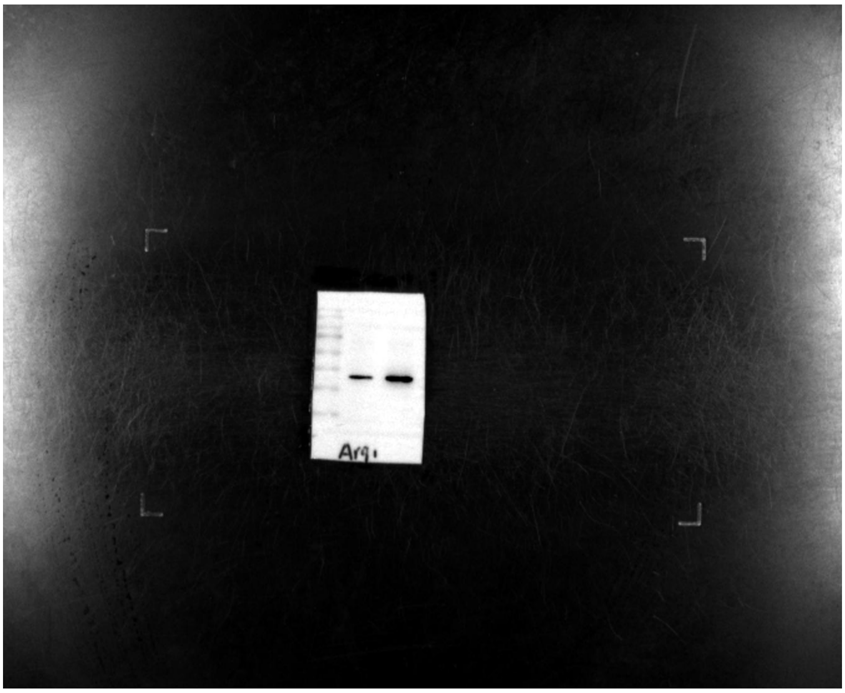

Full unedited gel/blot for Figure 3J CD16

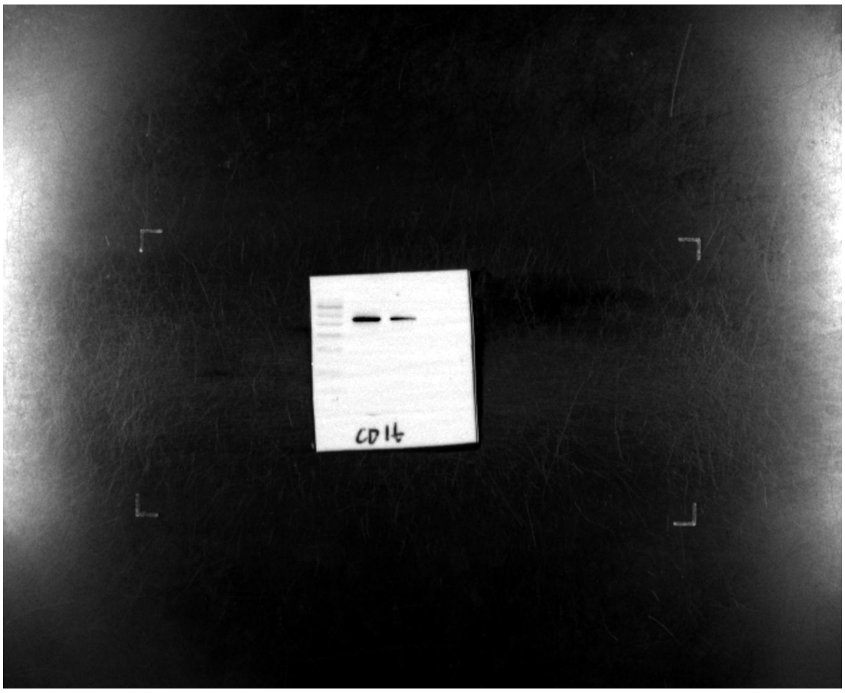

Full unedited gel/blot for Figure 3J GPX4

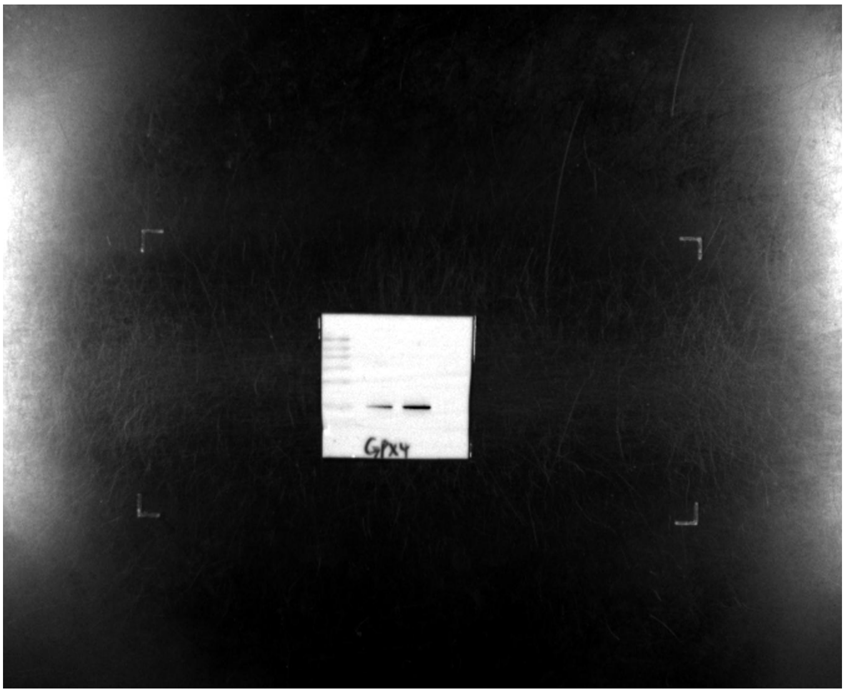

Full unedited gel/blot for Figure 3J INOS

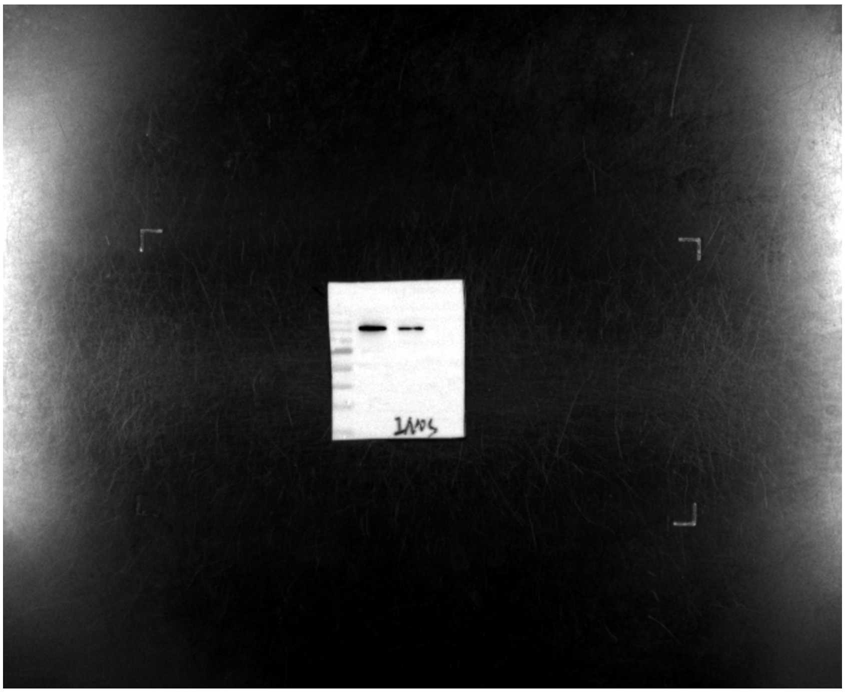

Full unedited gel/blot for Figure 3J XCT

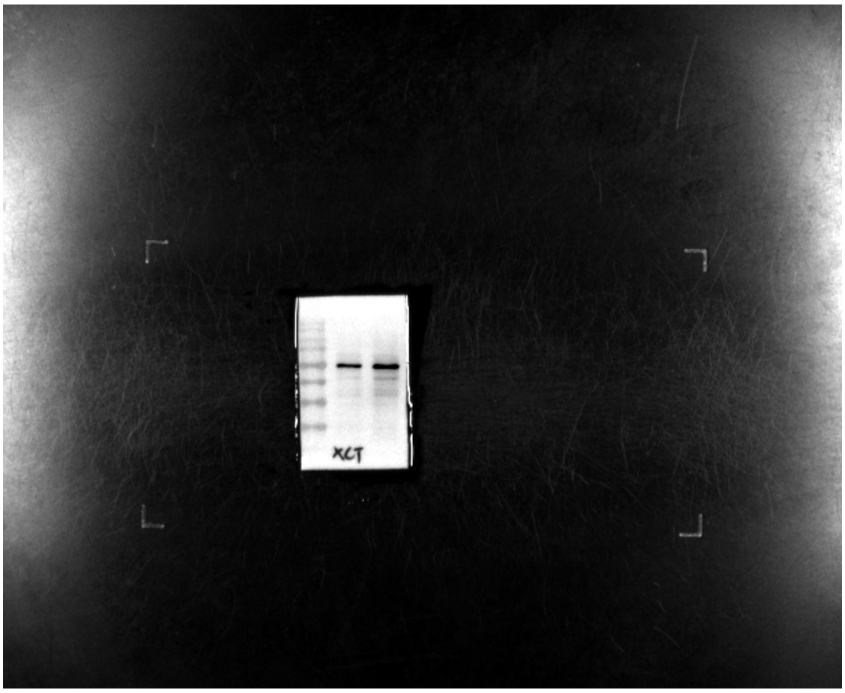

Full unedited gel/blot for Figure 4A GAPDH

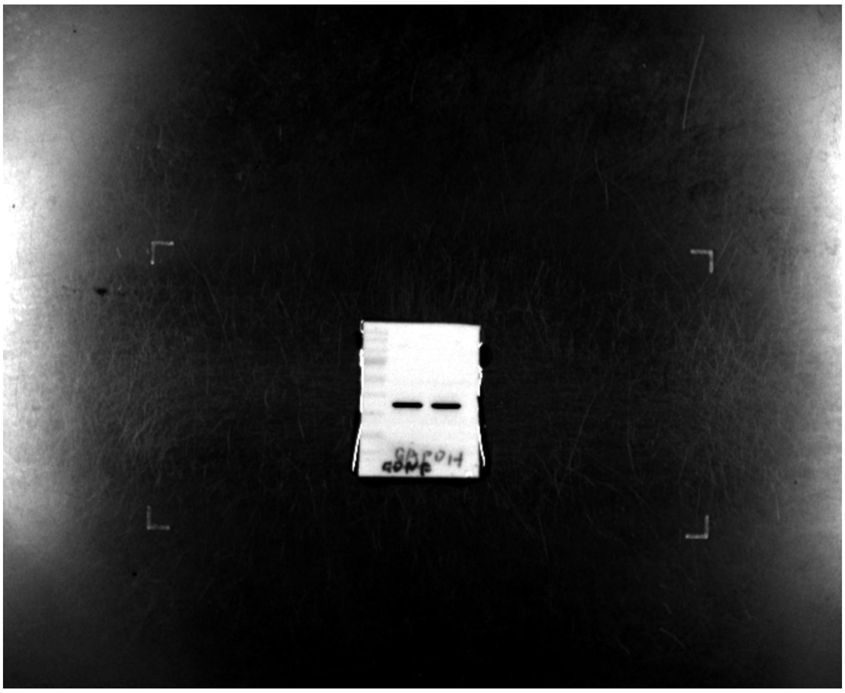

Full unedited gel/blot for Figure 4A GDNF

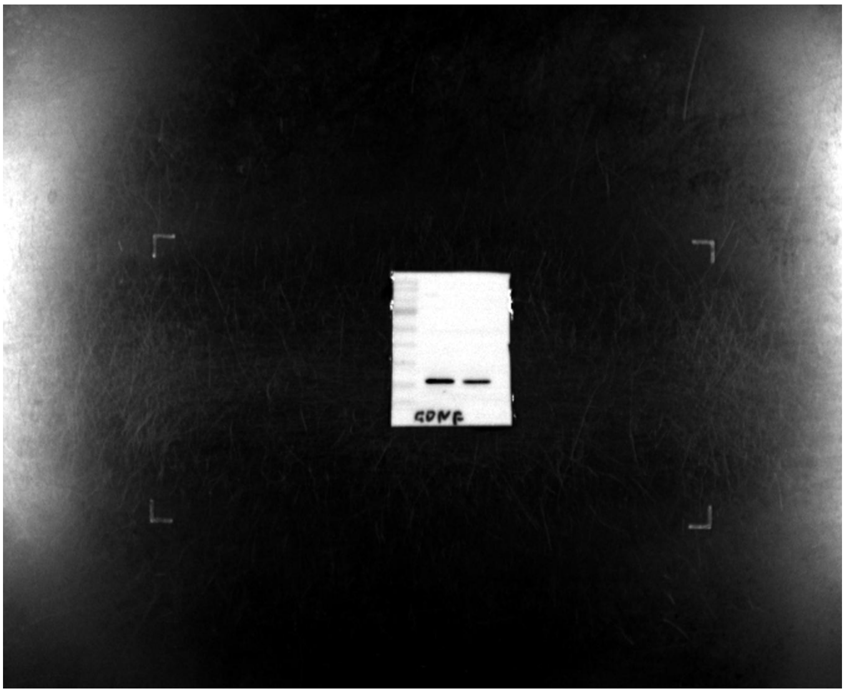

Full unedited gel/blot for Figure 4C H3K4me3

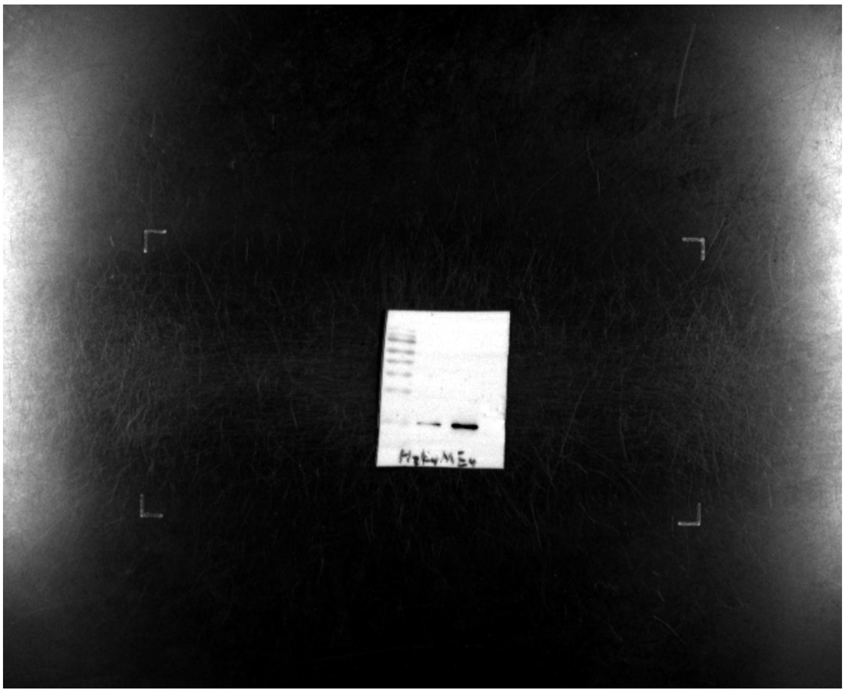

Full unedited gel/blot for Figure 4C Lamin A

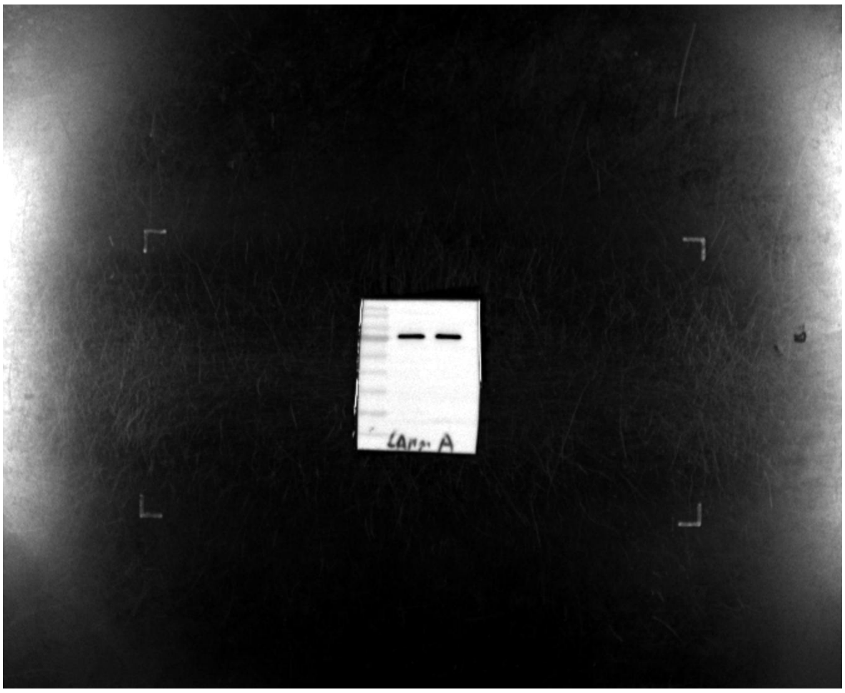

Full unedited gel/blot for Figure 4D GAPDH

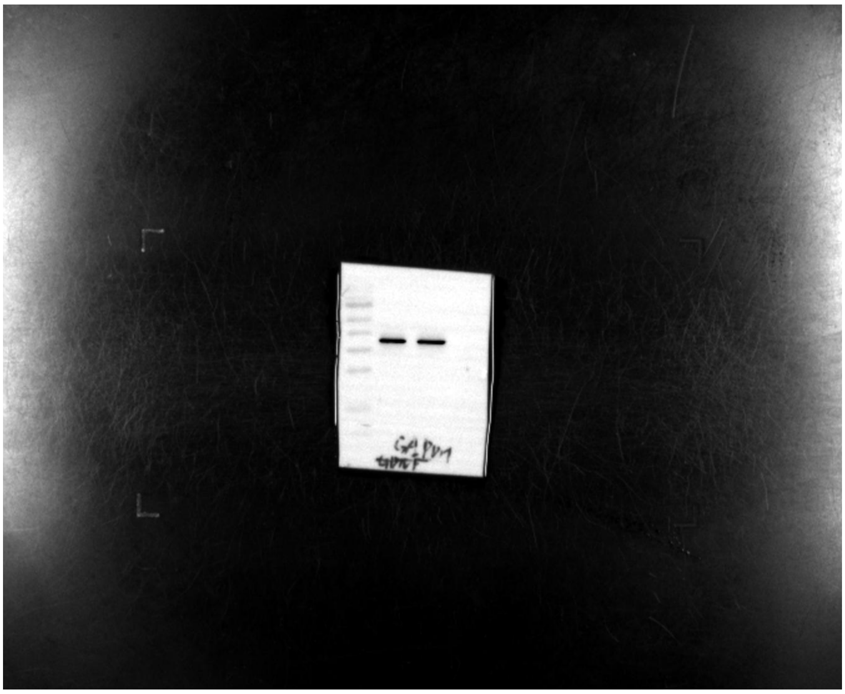

Full unedited gel/blot for Figure 4D GDNF

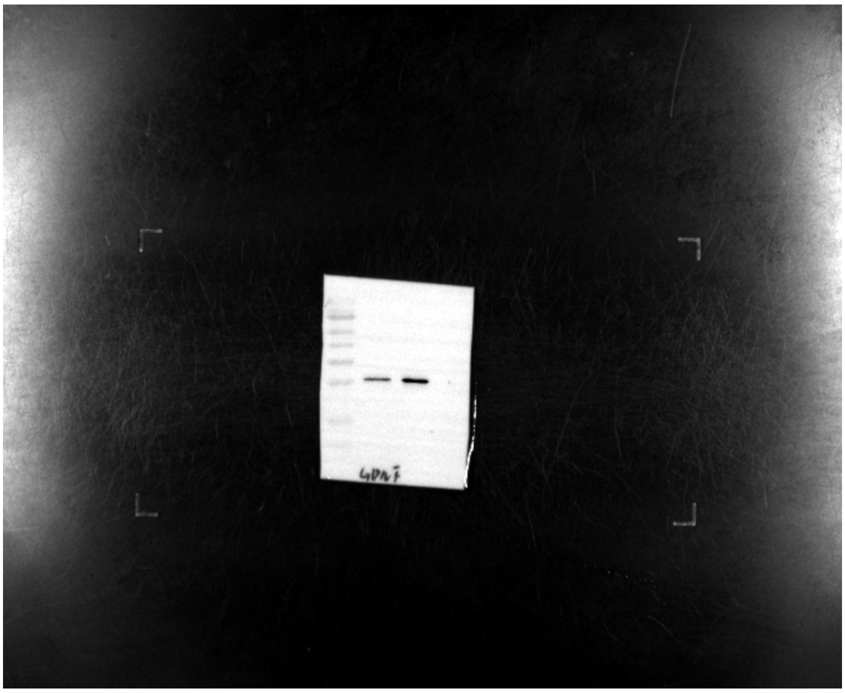

Full unedited gel/blot for Figure 4D PRMT8

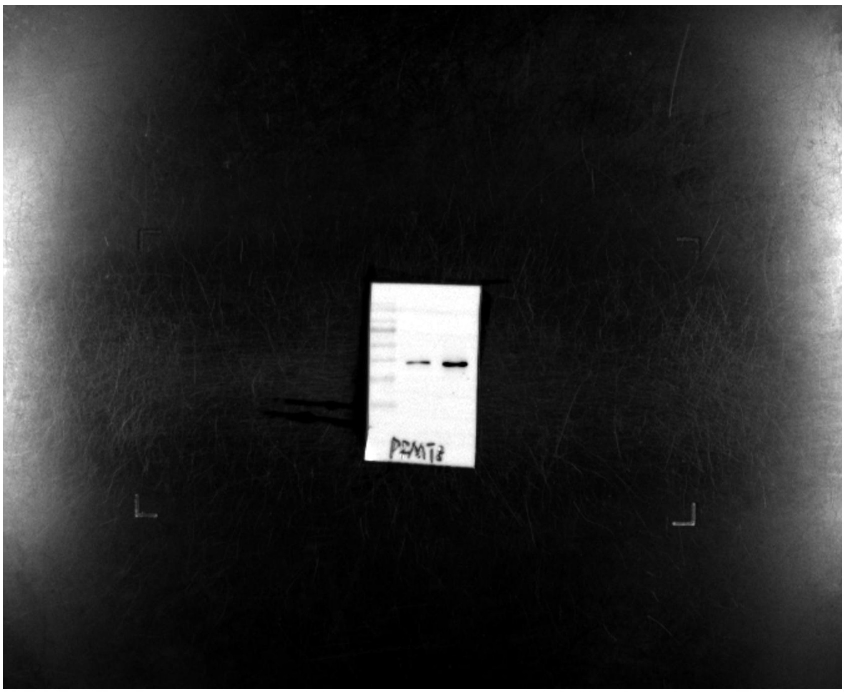

Full unedited gel/blot for Figure 5A GAPDH

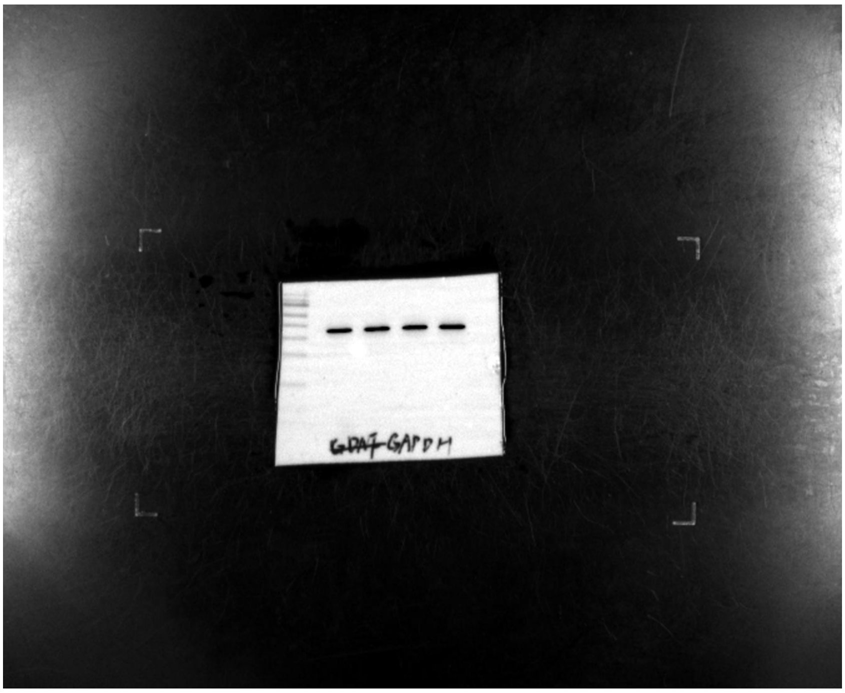

Full unedited gel/blot for Figure 5A GDNF

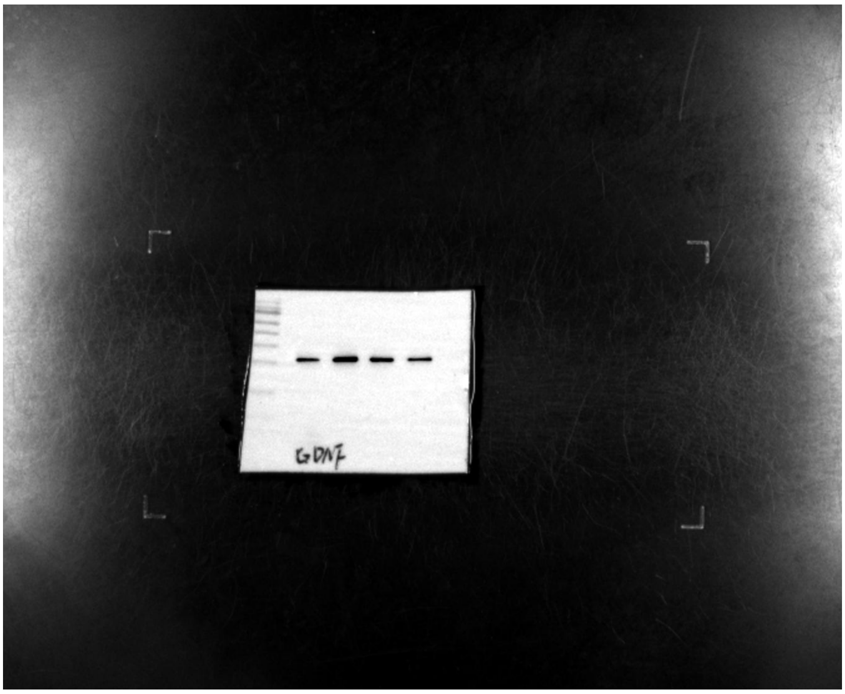

Full unedited gel/blot for Figure 5A PRMT8

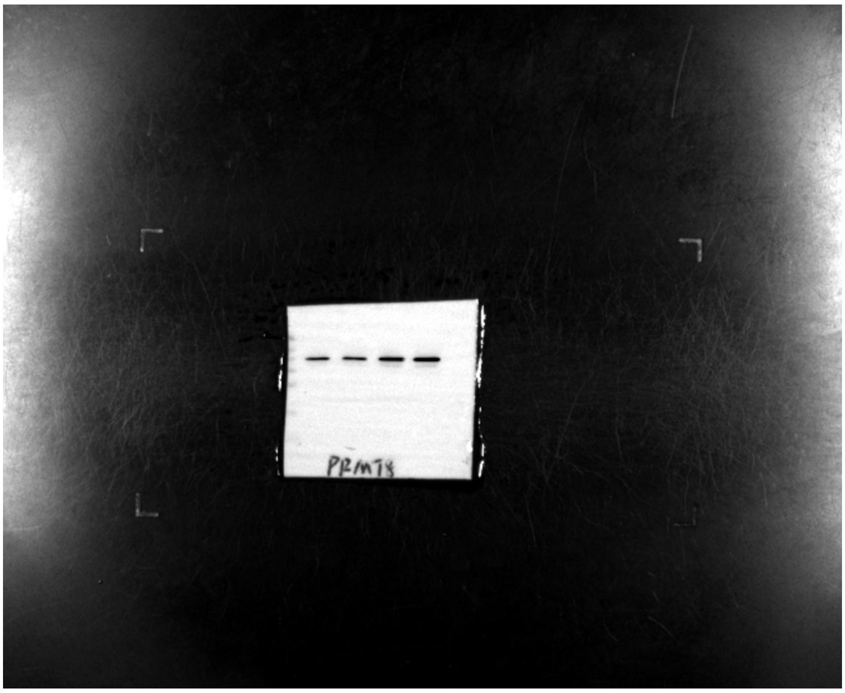

Full unedited gel/blot for Figure 5D GAPDH

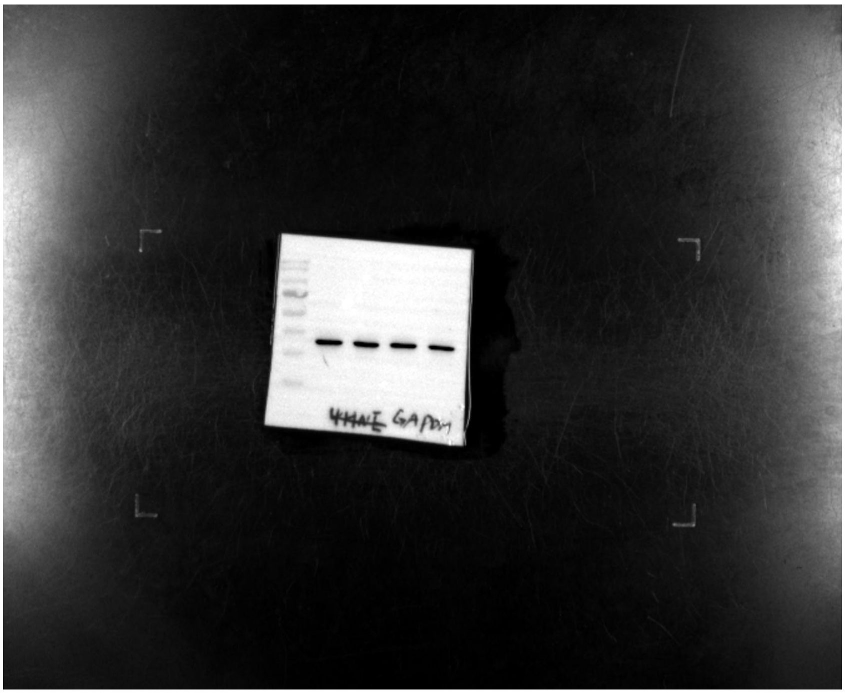

Full unedited gel/blot for Figure 5D 4HNE

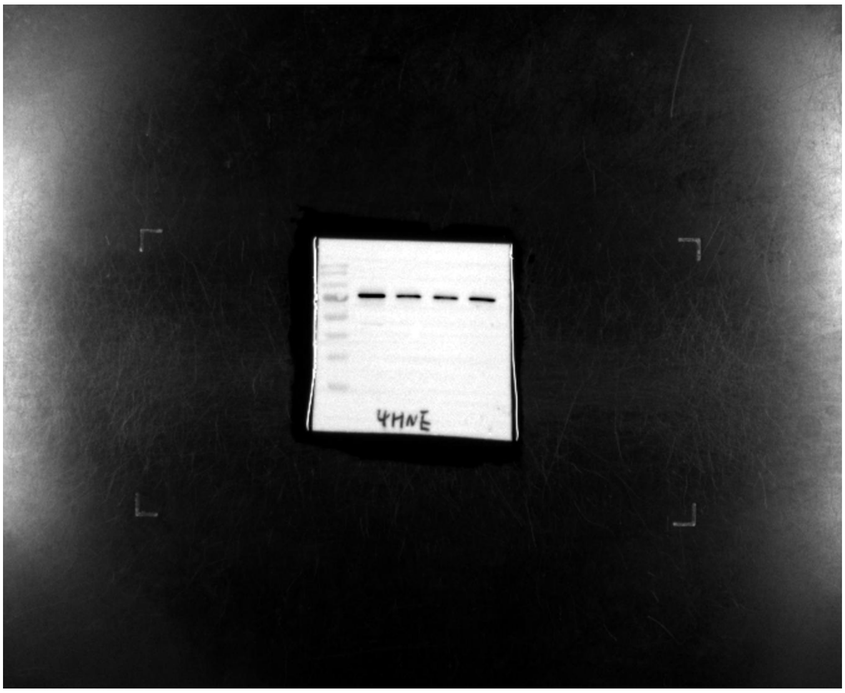

Full unedited gel/blot for Figure 5D HO-1

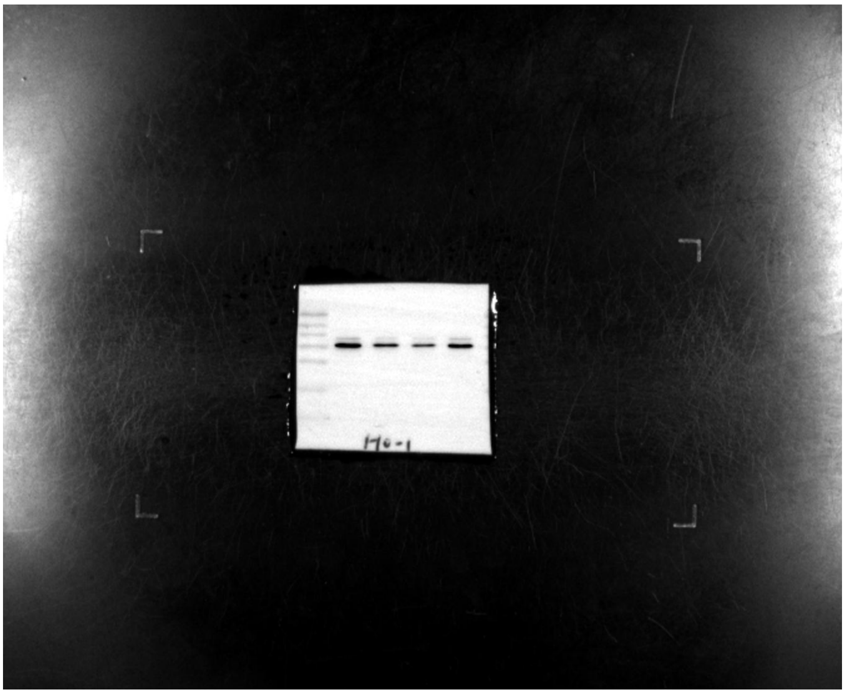

Full unedited gel/blot for Figure 5G GAPDH

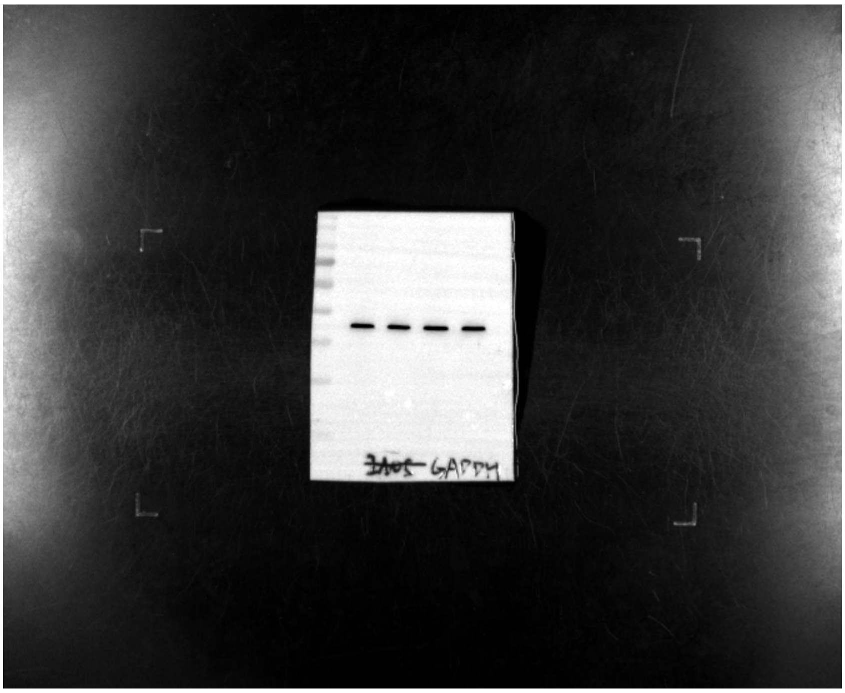

Full unedited gel/blot for Figure 5G Arg-1

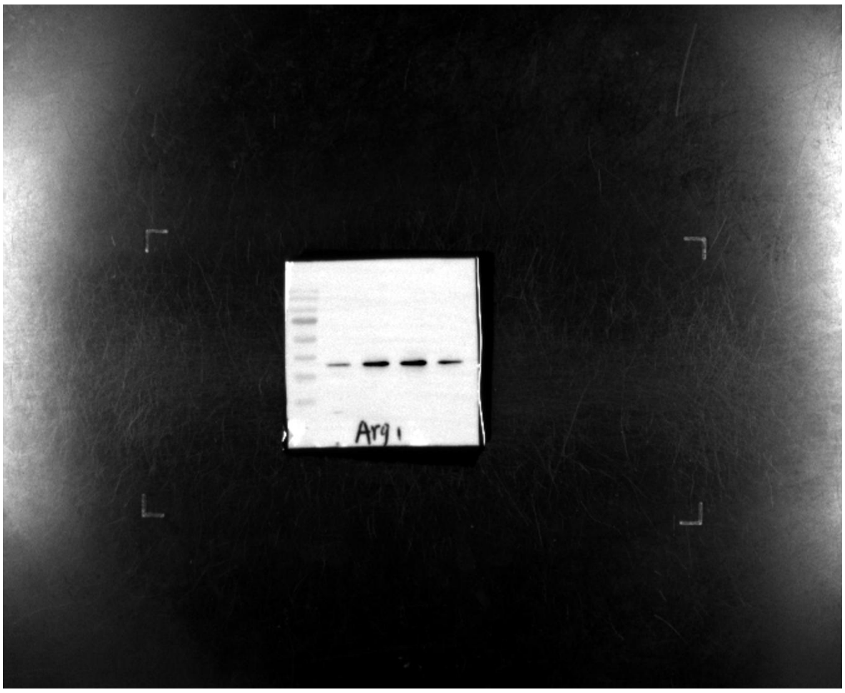

Full unedited gel/blot for Figure 5G INOS

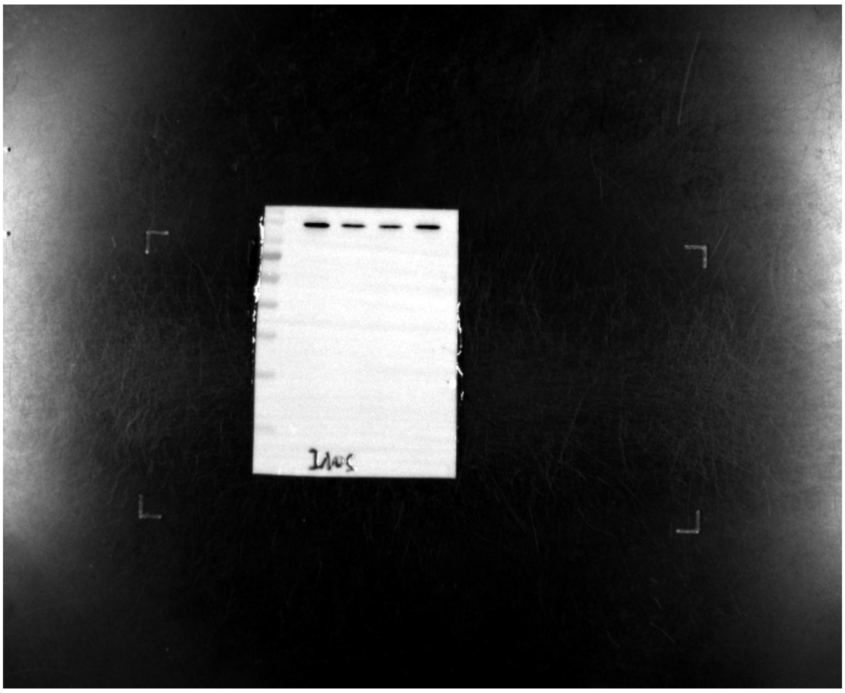

Full unedited gel/blot for Figure 6A GAPDH

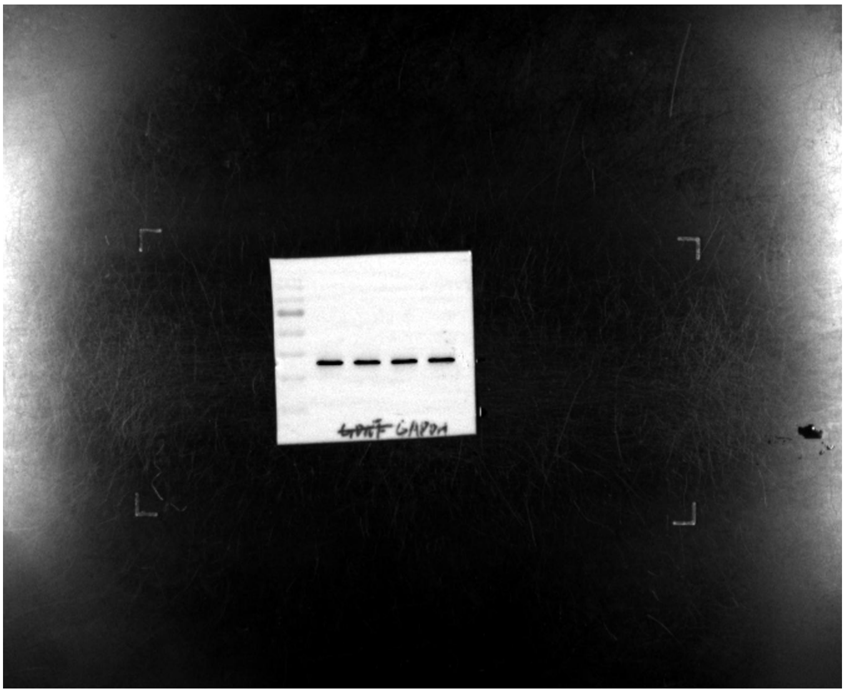

Full unedited gel/blot for Figure 6A GDNF

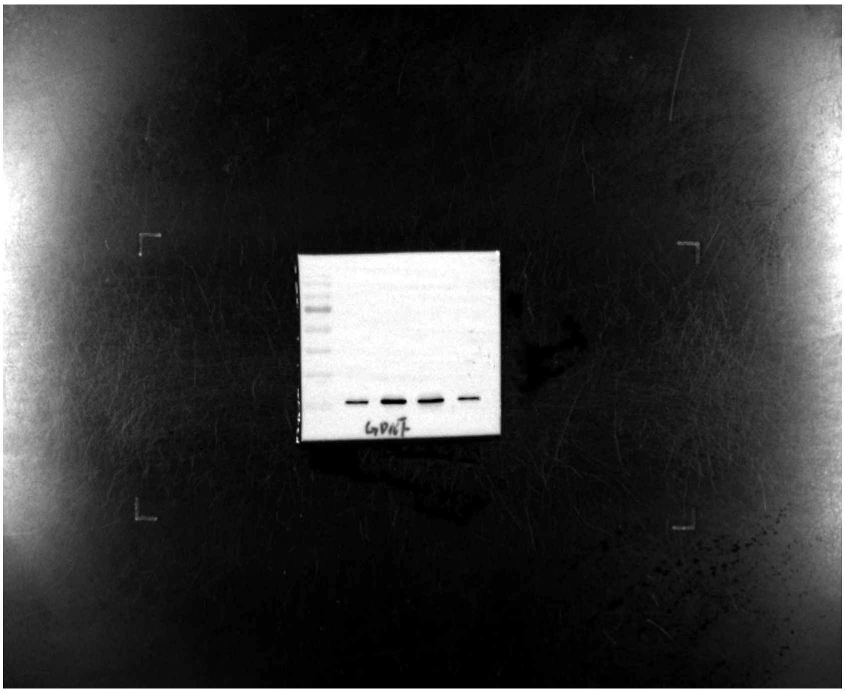

Full unedited gel/blot for Figure 6A PRMT8

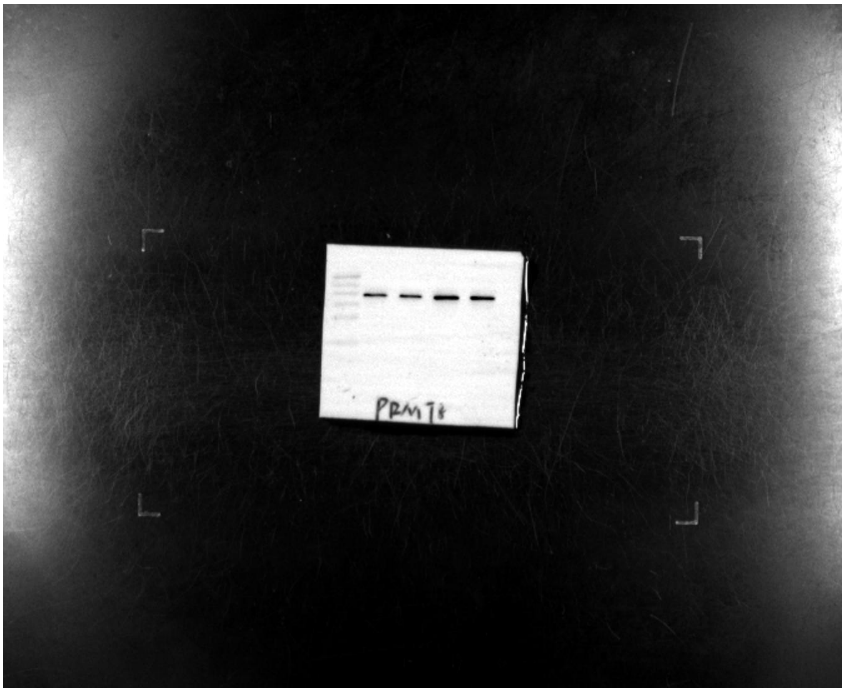

Full unedited gel/blot for Figure 6J GAPDH

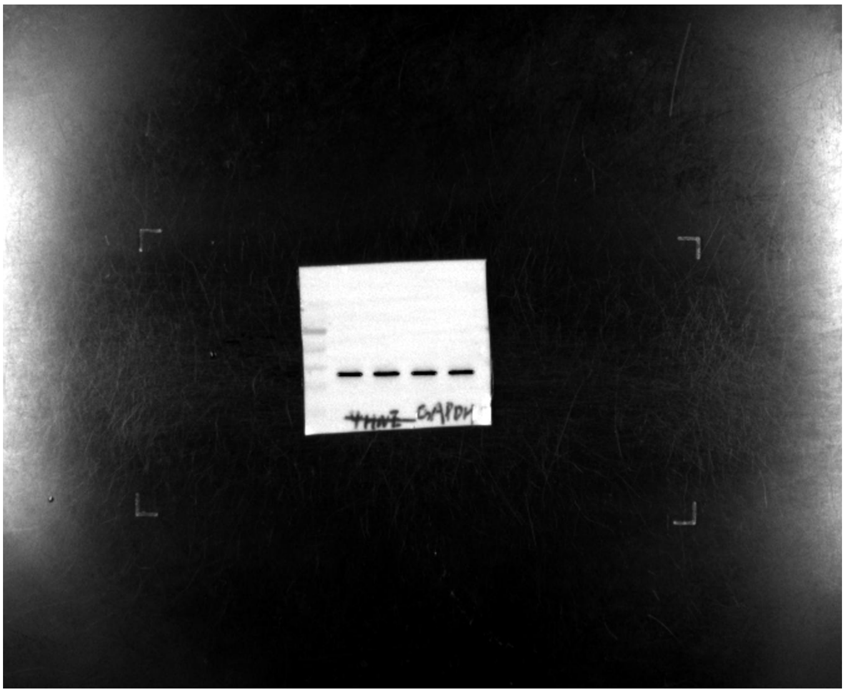

Full unedited gel/blot for Figure 6J 4-HNE

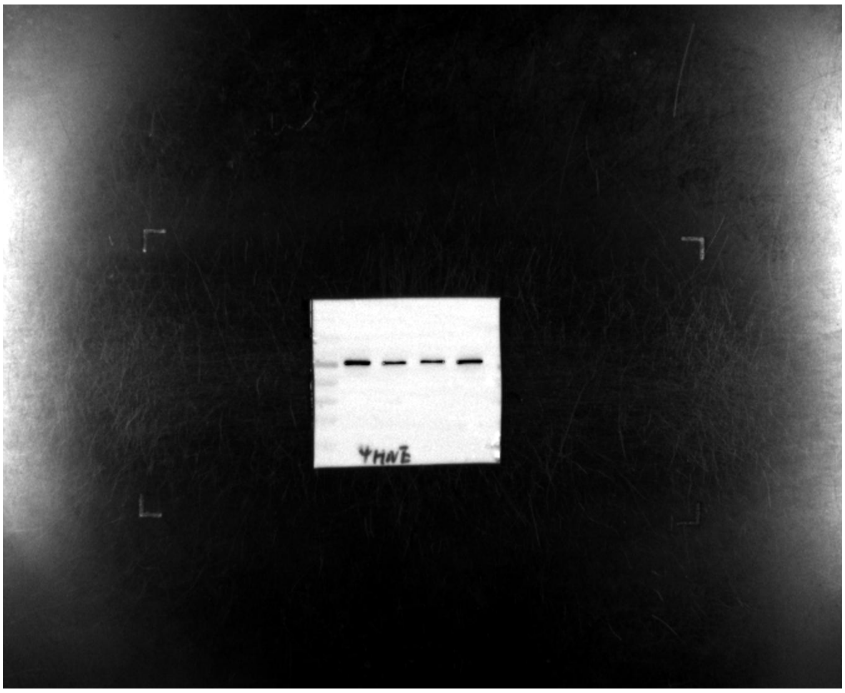

Full unedited gel/blot for Figure 6J GPX4

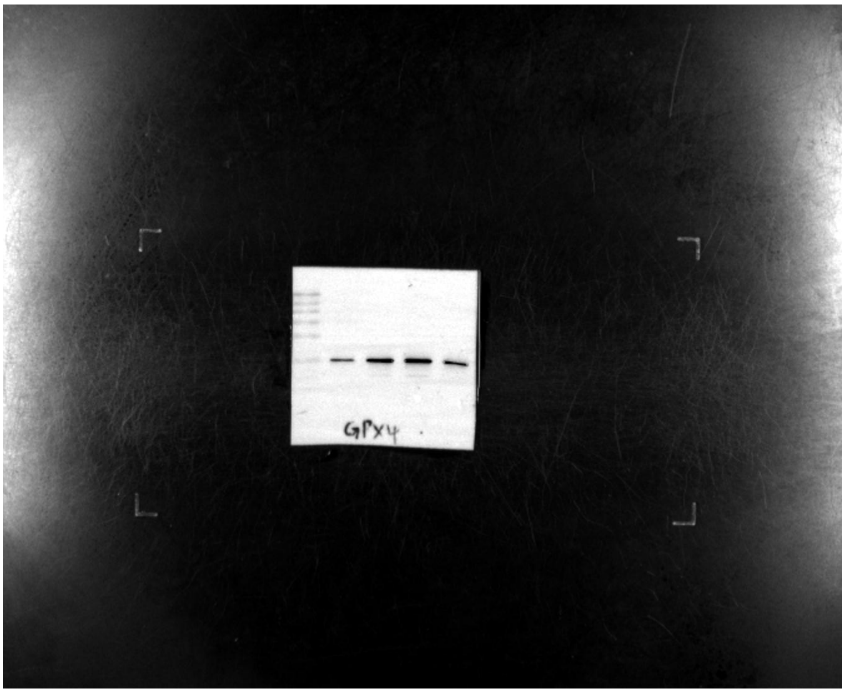

Full unedited gel/blot for Figure 6J XCT

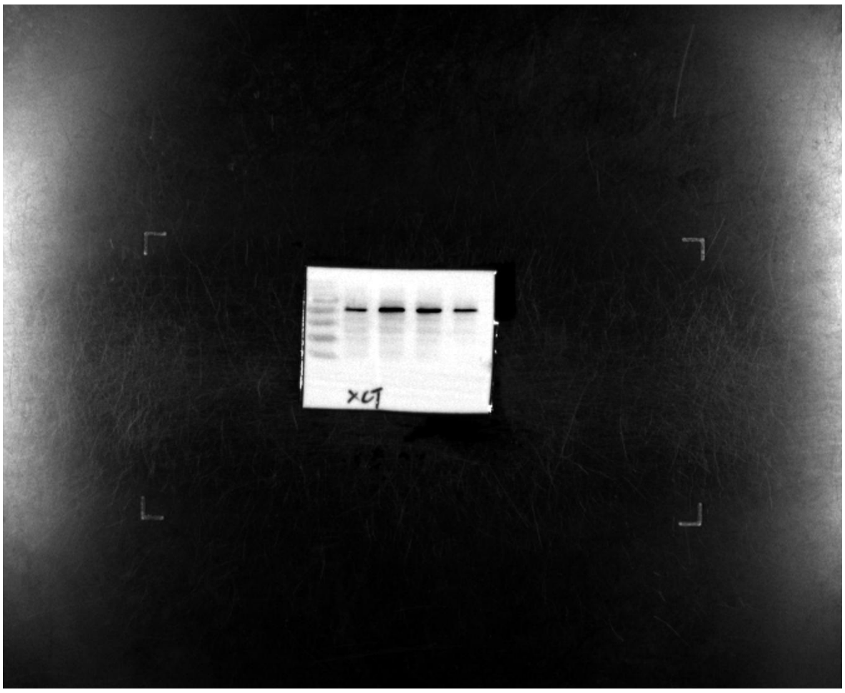

Full unedited gel/blot for Figure 6J GAPDH

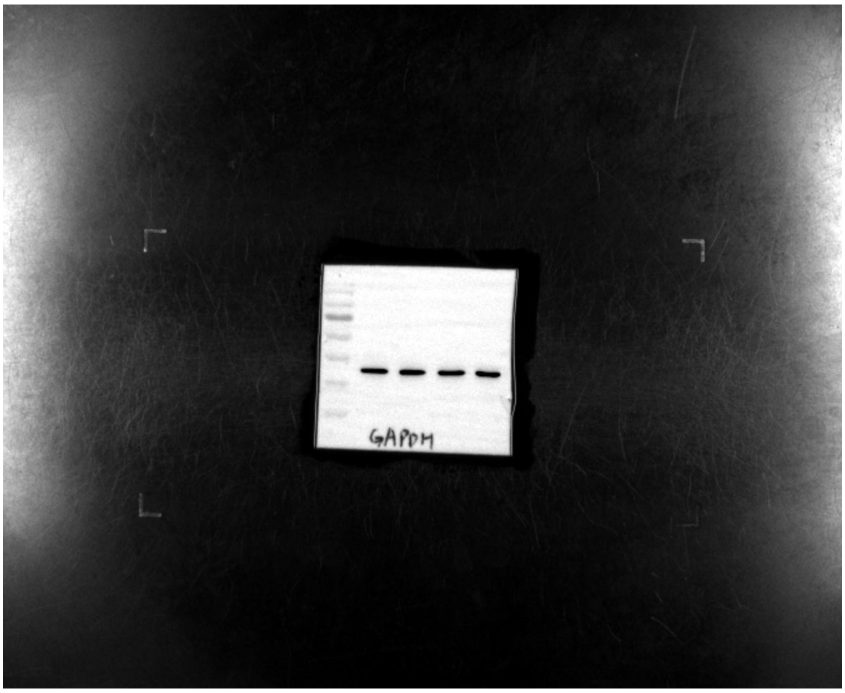

Full unedited gel/blot for Figure 6J Arg-1

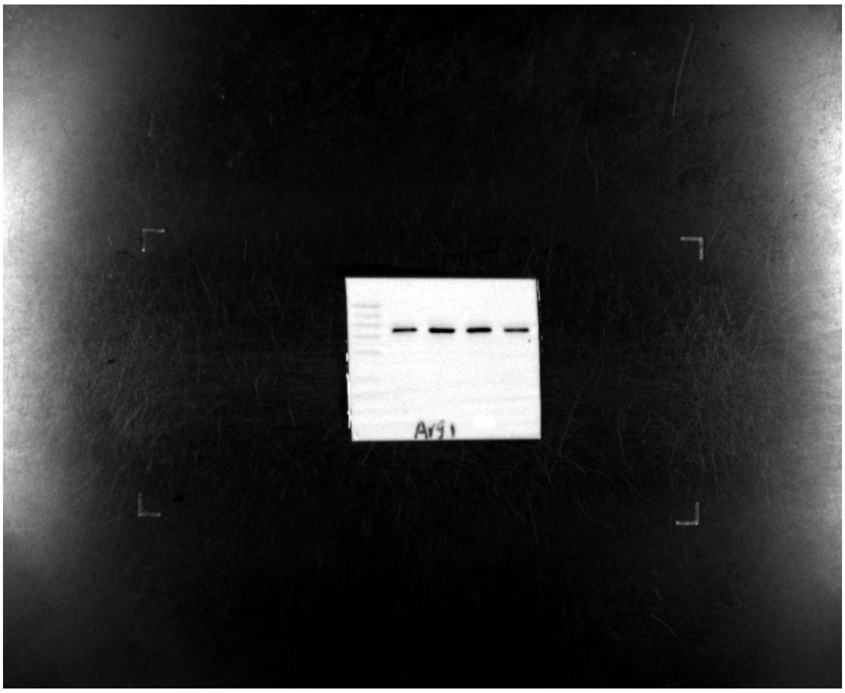

Full unedited gel/blot for Figure 6J CD16

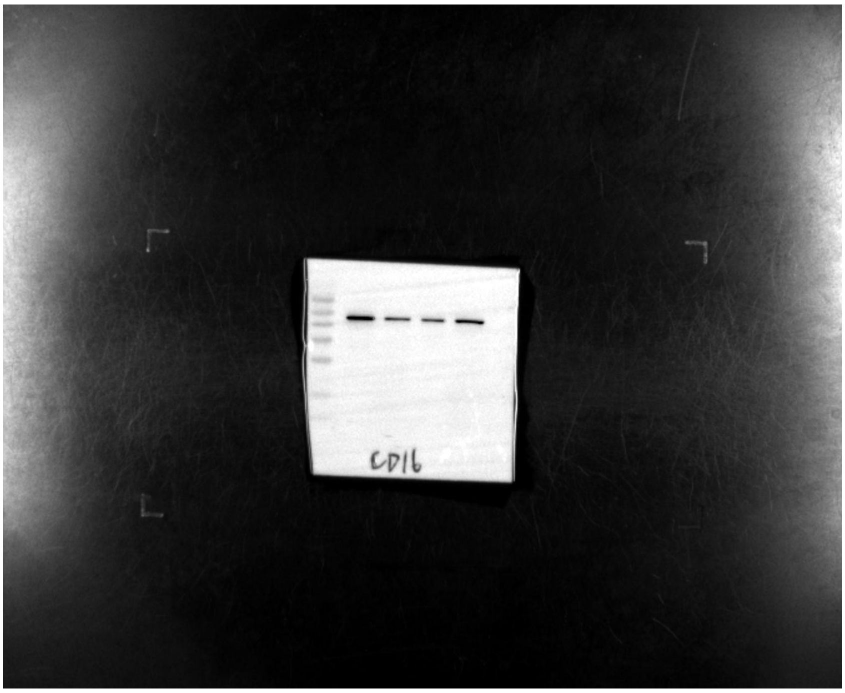

Full unedited gel/blot for Figure 6J INOS

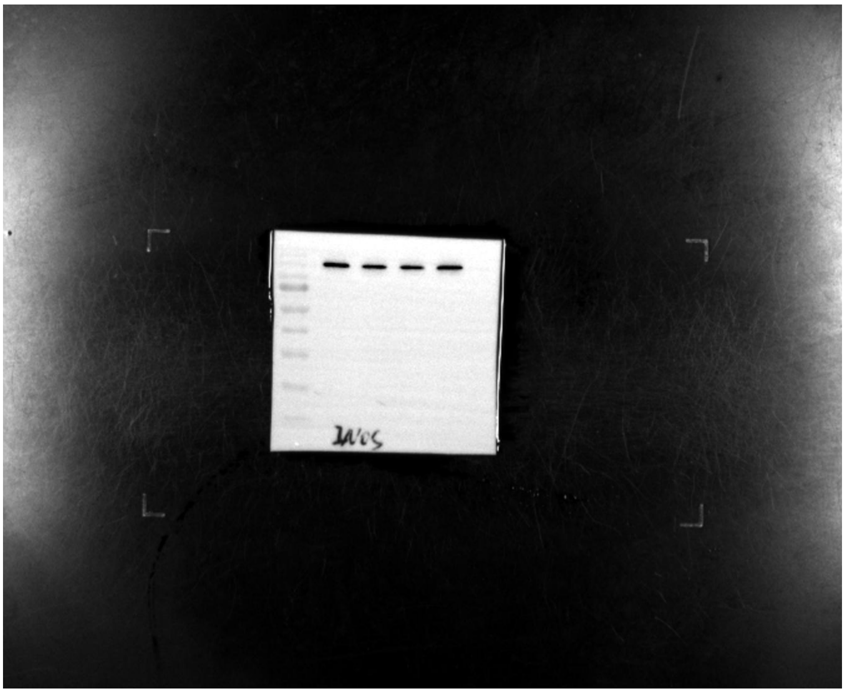

Supplement: Supplementary file 2 — Appendix S1 [file CNS-29-2145-s002.pdf]
